# Global Trends in the Proportion of Macrolide-Resistant *Mycoplasma pneumoniae* Infections: A Systematic Review and Meta-analysis

**DOI:** 10.1001/jamanetworkopen.2022.20949

**Published:** 2022-07-11

**Authors:** Kyunghoon Kim, Sungsu Jung, Mina Kim, Suyeon Park, Hyeon-Jong Yang, Eun Lee

**Affiliations:** 1 Department of Pediatrics, Seoul National University Bundang Hospital, Seongnam, Republic of Korea; 2 Department of Pediatrics, Seoul National University College of Medicine, Seoul, Republic of Korea; 3 Department of Pediatrics, Pusan National University Yangsan Hospital, Yangsan, Republic of Korea; 4 Department of Applied Statistics, Chung-Ang University, Seoul, Republic of Korea; 5 Department of Biostatistics, Soonchunhyang University College of Medicine, Seoul, Republic of Korea; 6 Department of Pediatrics, Soonchunhyang University Seoul Hospital, Soonchunhyang University College of Medicine, Seoul, Republic of Korea; 7 Department of Pediatrics, Chonnam National University Hospital, Chonnam National University Medical School, Gwangju, Republic of Korea

## Abstract

**Importance:**

The proportion of macrolide-resistant *Mycoplasma pneumoniae* (MRMP) infections has changed, and it differs according to geographical region.

**Objective:**

To analyze the global patterns, including the temporal trends, regional variations, and variant types, in the proportion of MRMP infections in this systematic review and meta-anaysis.

**Data Sources:**

PubMed, Cochrane Library, and Embase databases were searched for observational studies from inception to September 10, 2021.

**Study Selection:**

Observational studies reporting the proportion of MRMP infections were screened independently by 2 authors. The presence of MRMP infection was defined as any case of *M pneumoniae* infection positive for any variants associated with macrolide resistance identified using respiratory samples.

**Data Extraction and Synthesis:**

Data were extracted independently and in duplicate by 2 reviewers. The Preferred Reporting Items for Systematic Reviews and Meta-analyses (PRISMA) guideline was used. Random-effects meta-analyses were used to estimate the proportion of MRMP infections.

**Main Outcomes and Measures:**

The global patterns in the proportion of MRMP infections were estimated, and the temporal trends and variant types of MRMP infection with regional differences were investigated.

**Results:**

This study included 153 studies from 150 articles (27 408 samples in 26 countries) in the meta-analysis. The global patterns in the proportion of MRMP infections showed an increasing trend with regional differences. The proportion of MRMP infections was highest in the Western Pacific regions (53.4%; 95% CI, 47.4%-60.3%), followed by the South East Asian region (9.8%; 95% CI, 0.8%-100%), the region of the Americas (8.4%; 95% CI, 6.1%-11.6%), and the European region (5.1%; 95% CI, 3.3%-8.0%). The most commonly identified variant of MRMP infection was A2063G (96.8%; 95% CI, 95.8%-97.7%), followed by A2064G (4.8%; 95% CI, 3.5%-6.7%). The proportion of MRMP infections was the highest in studies including only children (37.0%; 95% CI, 29.8%-46.1%), followed by those including only adults (15.9%; 95% CI, 6.4%-39.7%) and those including both children and adults (16.7%; 95% CI, 10.1%-27.6%).

**Conclusions and Relevance:**

This study provides global trends in the proportion of MRMP infections and suggests that strategies to prevent the spread of MRMP infection and to treat MRMP infections are needed to decrease disease burden.

## Key Points

**Question:**

What are the temporal and regional trends in the proportion of macrolide-resistant *Mycoplasma pneumoniae* (MRMP) infections?

**Findings:**

In this systematic review and meta-analysis of 150 articles including 27 408 samples, the proportion of MRMP infections differed in temporal trends and variant types according to geographical region. The proportion of MRMP infections was highest in the Western Pacific regions, followed by the Southeast Asian region, the region of the Americas, and the European region.

**Meaning:**

This study suggests that prevention efforts are needed to decrease MRMP disease burden.

## Teaser

This systematic review and meta-analysis examines the global patterns, including temporal trends, regional variations, and variant types, in the proportion of macrolide-resistant *Mycoplasma pneumoniae* infections.

## Introduction

*Mycoplasma pneumoniae* is one of the most common causes of community-acquired pneumonia (CAP) in children, accounting for approximately 30% to 40% of cases.^1,2^ Some cases of *M pneumoniae* infection have been considered as a self-limiting disease, whereas other cases of *M pneumoniae* infection have led to poor clinical outcomes with serious complications.^3,4,5,6^ *Mycoplasma pneumoniae* lacks a cell wall and is thus resistant to antibiotics targeting the cell walls. Because antibiotics targeting cell walls are not an option for treating *M pneumoniae* infection, antibiotic treatment options for *M pneumoniae* infections include those that play a role in the disruption of protein synthesis (eg, macrolides and tetracyclines) and in the inhibition of DNA replication (eg, fluoroquinolones).^7^ However, tetracyclines and fluoroquinolones have limited use for children because of the lack of information on the safety in this population.^8^ Therefore, macrolides have been the first choice for treating *M pneumoniae* infection in children.

However, accumulating evidence suggests that the prevalence of refractory *M pneumoniae* pneumonia has been increasing.^7,9^ Refractory *M pneumoniae* pneumonia has been associated with difficult-to-treat *M pneumoniae* infection, increased long-term complications, and increased medical costs with decreased quality of life.^10,11^ Macrolide resistance of *M pneumoniae* is one of the possible causes of refractory *M pneumoniae* pneumonia, although asymptomatic cases of macrolide-resistant *M pneumoniae* (MRMP) infection have rarely been reported.^12^ Macrolide resistance is caused by variants in the V region of the 23S rRNA gene, which codes for the binding site of macrolides in the *M pneumoniae* ribosome.^7^ Debate exists as to whether there are differences in clinical features, including the severity of pneumonia, between patients with MRMP infection and those with macrolide-sensitive *M pneumoniae* (MSMP) infecton. Some studies have reported no differences in clinical characteristics between the 2 groups,^13,14,15^ whereas other studies have reported longer fever duration, more severe clinical courses, and increased risk of intensive care unit admission among patients with MRMP infection compared with patients with MSMP infection.^9,16,17,18^ The detection of MRMP can occur during treatment with macrolides among patients initially infected with MSMP.^19^ Coinfection cases with MSMP and MRMP have rarely been reported,^19,20^ indicating the advent of MRMP due to macrolide treatment for *M pneumoniae* infection.

The proportion of MRMP infections varies by geographical region.^21,22,23^ In addition, there is a difference in the proportion of MRMP infections according to time period, and the proportion of specific MRMP variant types differs according to region and time period. However, to date, there have been no studies on the global patterns in the proportion of MRMP infections, to our knowledge. The primary outcome of this study was to identify the global trends in the proportion of MRMP infections. The secondary outcomes were to evaluate the proportion of MRMP infections according to temporal trends, regional variations, and variant types.

## Methods

### Study Selection

**Figure 1.**
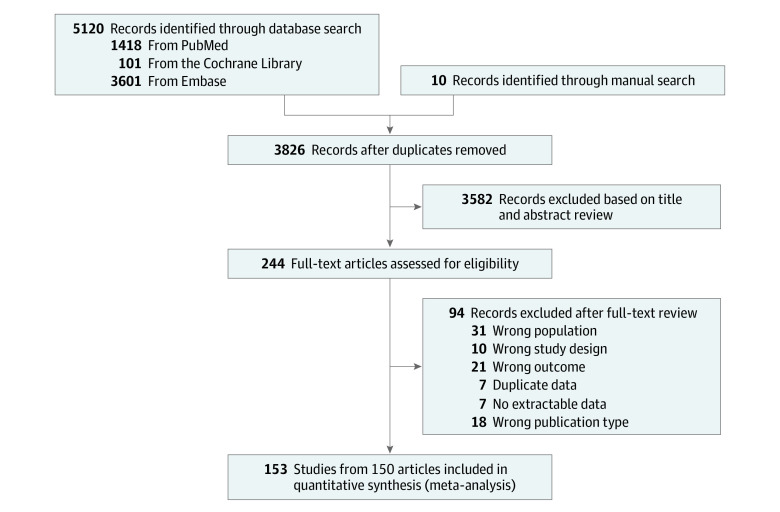
PRISMA Flow Diagram

Two reviewers (K.K. and S.J.) independently screened articles for eligibility, and disagreements about whether specific articles should be included in our analyses were resolved on a consensus basis or by consulting a third reviewer (H.-J.Y. or E.L.) when consensus was not reached. Studies that investigated the proportion of MRMP infections using polymerase chain reaction (PCR) were included without regard to the age of patients in the study population. We defined MRMP infection as any case of *M pneumoniae* infection positive for any variants associated with macrolide resistance. Studies that aimed to validate the newly developed diagnostic method (such as Lightmix) were excluded, even though they included a proportion of MRMP infections. We recorded the selection process in sufficient detail to complete the Preferred Reporting Items for Systematic Reviews and Meta-analyses (PRISMA) flow diagram (Figure 1).

### Literature Search Strategy

We conducted the systematic review and meta-analysis in accordance with the PRISMA reporting guideline.^24^ We searched the observational studies in the PubMed, Cochrane Library, and Embase databases by using a combination of search terms related to *M pneumoniae*, prevalence, resistance, and macrolides (eAppendix in the Supplement). No language restrictions were applied. Studies published until September 10, 2021, were included. This meta-analysis study was not registered.

### Data Extraction and Quality Assessment

In the studies examined here, the diverse types of variants associated with MRMP infection were reported. Data pertaining to secondary outcomes were extracted when available. Data on study author, publication year, study periods, study country, participants’ age, participants’ sex, detection methods of macrolide resistance of *M pneumoniae*, number of study population, types of respiratory tract infection (RTI; eg, upper RTI or pneumonia), proportion of MRMP infections, and variant types of MRMP infection were extracted. We could not obtain information on some of these items depending on the studies.

Three reviewers (K.K., S.J., and E.L.) independently assessed the quality of the included studies for risk of bias using the Strengthening the Reporting of Observational Studies in Epidemiology (STROBE) reporting guideline in 5 items: sample population, sample size, participation rate, outcome assessment, and analytical methods to control for bias (eTable 1 in the Supplement).^25,26^ We resolved disagreement by discussion with a fourth author (H.-J.Y.) and other authors who were not involved in assessment of the quality of studies for bias risk in the first step.

### Statistical Analysis

We used random-effects meta-analyses to calculate the proportion of MRMP infections. The *I*^2^ statistic was used to assess heterogeneity in the results of individual studies, and an *I*^2^ statistic greater than 50% was used as a threshold indicating significant heterogeneity. The Egger test was used to assess potential publication bias. For studies that were performed for more than 2 years without proportion information provided by year, we used the midpoint of the study period owing to heterogeneity in the study year and study period. We conducted sensitivity analyses in which overall summary estimates were restricted to studies of CAP due to *M pneumoniae* in children. We performed subgroup analyses by time period, World Health Organization (WHO) geographical regions, variant types associated with macrolide resistance of *M pneumoniae*, age groups (children, adults, and studies including both children and adults), and types of RTI (any RTI vs CAP). The Mann-Kendall trend test was used to identify the consistently increasing or decreasing trend in the proportion of MRMP infections over time.

## Results

### Study Selection

**Figure 2.**
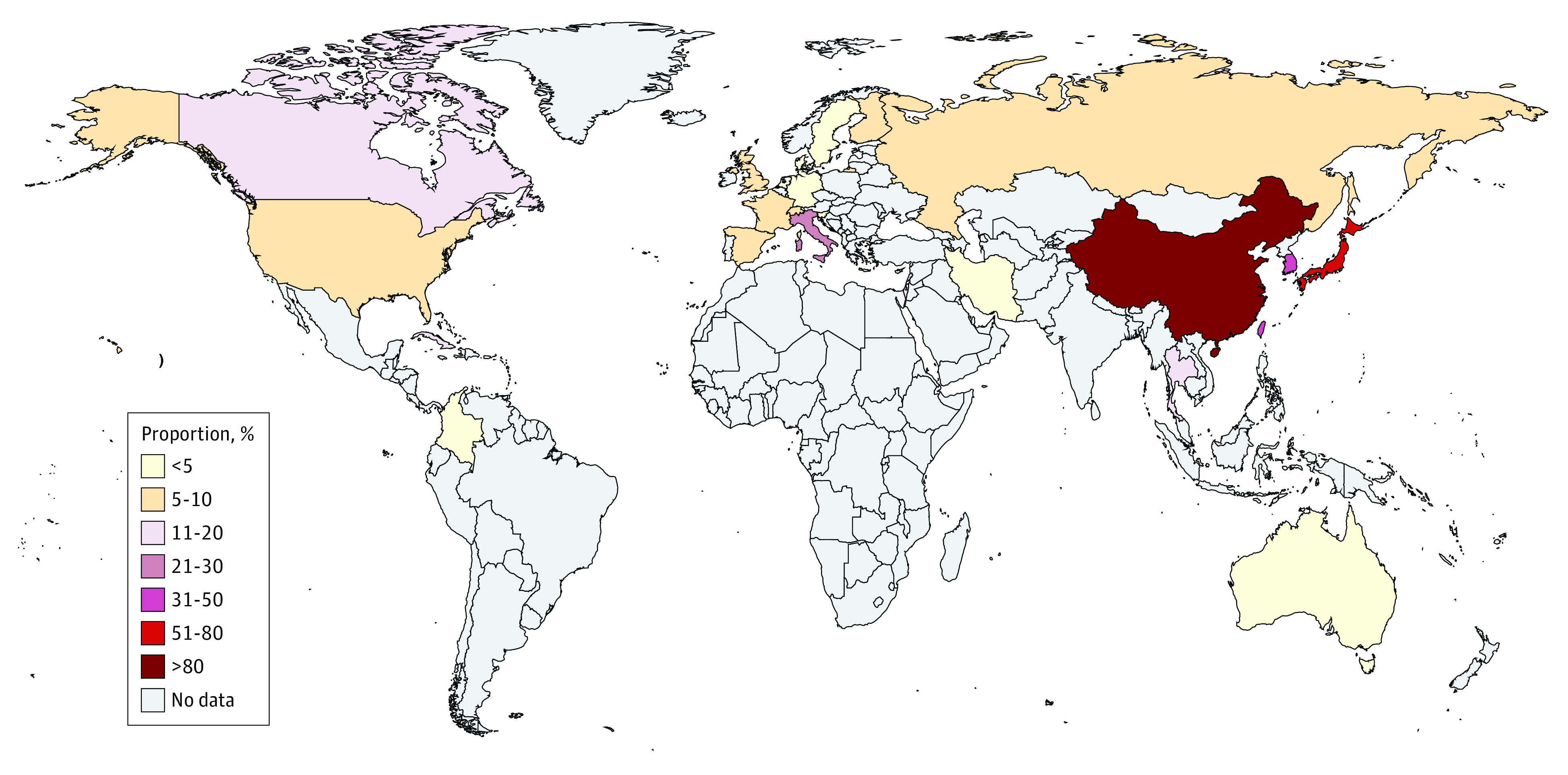
Map of the Proportion of Macrolide-Resistant *Mycoplasma pneumoniae* Infections by Country

After we adopted the eligibility criteria, 244 articles underwent full-text review. A total of 94 articles were excluded from this initial step (Figure 1). A total of 150 articles, involving 27 408 samples in 26 countries between inception and September 10, 2021, were included. Of the 150 articles, 3 included information on the proportion of MRMP infections from 2 countries in different WHO regions (France and Israel^27^; Australia and China^28^; and Japan and the US^29^). Because the proportion of MRMP infections varies considerably from one region to the next (Figure 2), we opted to separate the independent information on the proportion of MRMP infections in each country to avoid any potential confusion between the number of included articles and the number of studies on the proportion of MRMP infections in this systematic review and meta-analysis. The quality of the studies included ranged from 5 to 9 (high scores in each item indicate low risk; eTable 2 in the Supplement). The list and detailed characteristics of the 150 included articles are provided in eTable 3 in the Supplement.

### Study Characteristics

Of the 153 eligible data sets from 150 articles, 123 (80.4%) were retrospective data, and 30 (19.6%) included prospective data. A total of 84 studies (54.9%) were performed for children, 15 (9.8%) were performed for adults, and 35 (22.9%) included both children and adults (Table 1). However, 19 studies (12.4%) did not note the ages of the included population. The number of studies stratified by country is summarized in eTable 4 in the Supplement. Studies were performed most commonly in the Western Pacific region (103 [67.3%]), followed by the European region (31 [20.3%]), the region of the Americas (14 [9.2%]), the Southeast Asian region (3 [2.0%]), and the Eastern Mediterranean region (2 [1.3%]) (Table 1). We could not find published data from any countries in the WHO African region.

**Table 1.** Characteristics of the Included Studies and the Proportion of MRMP Infections

Variable	Studies, No.	Participants, No./total No.	Proportion of MRMP infections, % (95% CI)	*P* value for χ^2^ subgroup difference
WHO geographical regions				
Region of the Americas	14	163/2269	8.4 (6.1-11.6)	<.001
Eastern Mediterranean	2	1/117	1.4 (0.3-7.0)	
European	31	144/4414	5.1 (3.3-8.0)	
Southeast Asian	3	47/301	9.8 (0.8-100)	
Western Pacific	103	12 634/20 307	53.4 (47.4-60.3)	
WHO regions				
Western Pacific	103	12 634/20 307	53.4 (47.4-60.3)	<.001
Non-Western Pacific	50	355/7101	6.3 (4.5-8.6)	
Variant types				
A2063G	116	9163/9745	96.8 (95.8-97.7)	<.001
A2064G	88	188/9745	4.8 (3.5-6.7)	
Others	82	394/9745	6.3 (4.4-9.1)	
MRMP in studies with information on variant types	131	9745/27 408	26.7 (21.4-33.3)	
Gross domestic product				
Low	101	7680/19 334	20.0 (15.6-25.7)	<.001
Middle	45	5256/7587	60.9 (48.5-76.6)	
High	7	53/487	6.1 (1.6-23.8)	
Age group				
Children	84	8934/17 191	37.0 (29.8-46.1)	.007
Adults (≥19 y)	15	1727/3529	15.9 (6.4-39.7)	
Children and adults	35	1646/4804	16.7 (10.1-27.6)	
NA	19	682/1884	20.4 (11.0-37.8)	
Types of RTI				
CAP	65	5422/8939	38.8 (30.3-49.6)	.006
RTI	59	5121/11 997	23.0 (16.4-32.4)	
NA	29	2446/6472	17.8 (10.5-30.4)	

Abbreviations: CAP, community-acquired pneumonia; MRMP, macrolide-resistant *Mycoplasma pneumoniae*; NA, not applicable; RTI, respiratory tract infection; WHO, World Health Organization.

### Proportion of MRMP Infections by Time and Region

**Figure 3.**
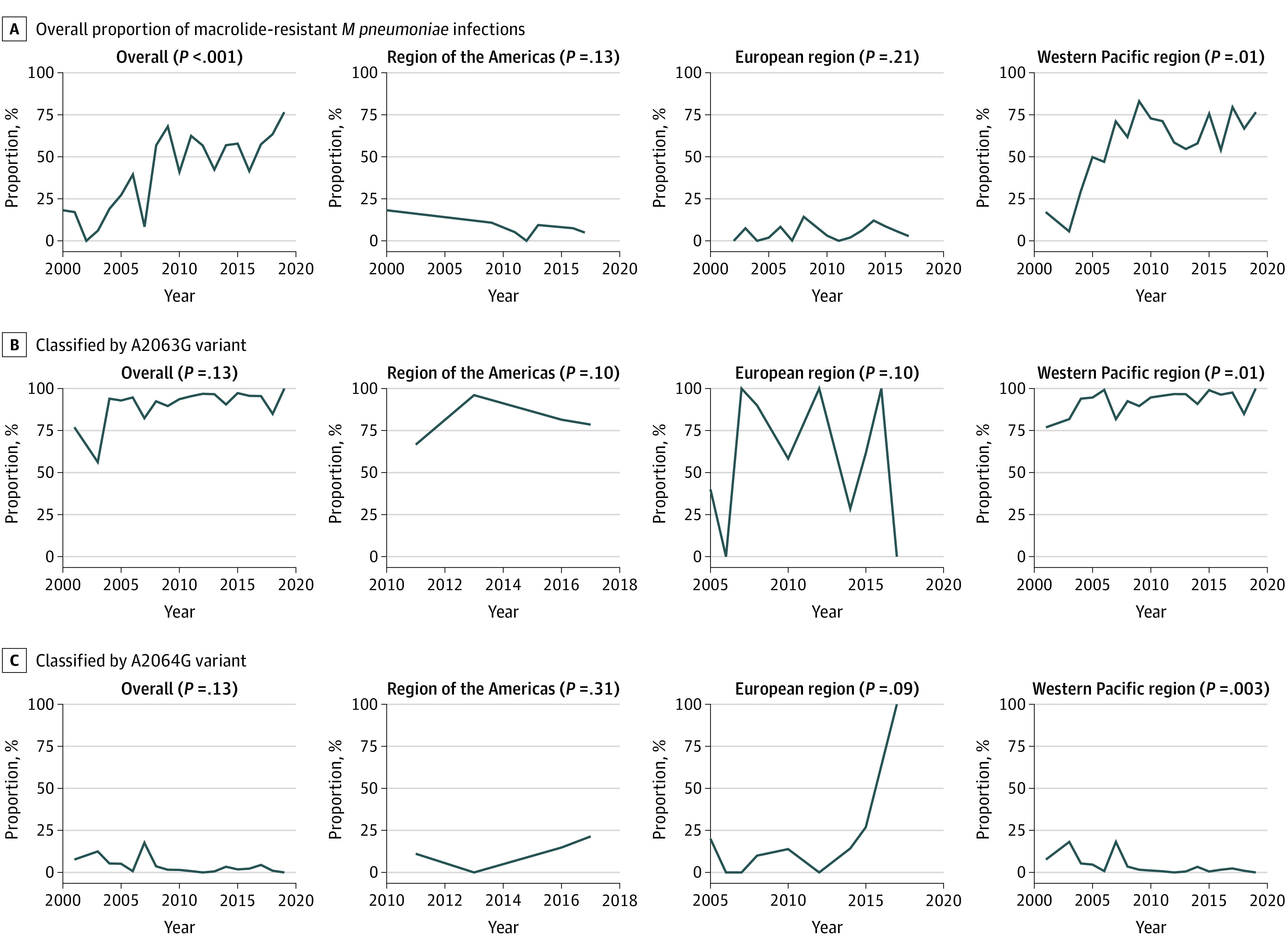
Proportion of Macrolide-Resistant *Mycoplasma pneumoniae* Infections Worldwide

The worldwide proportion of MRMP infections increased from 18.2% in 2000 to 41.0% in 2010 to 76.5% in 2019 (*P* < .001 for trend; Figure 3A; Table 2). When the proportion of MRMP infections was classified by WHO regions, a significant increasing trend was observed in the Western Pacific region (from 17.1% in 2001 to 71.2% in 2011 to 76.5% in 2019; *P* = .01 for trend). However, the proportion of MRMP infections did not significantly change over time in other WHO regions. When classified by variant types, including the A2063G and A2064G variants that are associated with MRMP infections, the increasing trend of MRMP infections over time was not significant except in the Western Pacific region (Figure 3B and C).

**Table 2.** Proportion of MRMP Infections by Year of Testing in Each WHO Region

Year of testing	MRMP Infections, %			
	Overall	Region of the Americas	European region	Western Pacific region
2000	18.2	18.2	NA	NA
2001	17.1	NA	NA	17.1
2002	0	NA	0	NA
2003	6.1	NA	7.5	5.6
2004	19.1	NA	0	29.4
2005	27.3	NA	1.9	49.8
2006	39.5	NA	8.3	47.0
2007	8.4	NA	0.2	71.1
2008	56.8	NA	14.3	61.8
2009	68.0	10.8	NA	83.0
2010	41.0	NA	3.1	72.8
2011	62.4	5.2	0	71.2
2012	56.7	0	2.0	58.5
2013	42.4	9.4	6.2	54.6
2014	56.9	NA	12.1	58.0
2015	57.8	NA	8.6	75.6
2016	41.5	7.5	5.6	54.1
2017	57.4	4.9	2.8	79.5
2018	63.4	NA	NA	66.8
2019	76.5	NA	NA	76.5

Abbreviations: MRMP, macrolide-resistant *Mycoplasma pneumoniae*; NA, not applicable; WHO, World Health Organization.

The highest proportion of MRMP infections was observed in the Western Pacific region (53.4%; 95% CI, 47.4%-60.3%), followed by the South East Asian region (9.8%; 95% CI, 0.8%-100%), the region of the Americas (8.4%; 95% CI, 6.1%-11.6%), the European region (5.1%; 3.3%-8.0%), and the East Mediterranean region (1.4%; 0.3%-7.0%) (eTable 4 in the Supplement). Within the Western Pacific region, the proportion of MRMP infections was highest in China (79.5%; 95% CI, 74.6%-84.8%), followed by Japan (47.3%; 95% CI, 38.9%-57.5%) and Taiwan (32.4%; 95% CI, 17.1%-61.2%) and was lowest in Australia (1.5%; 95% CI, 0.2%-11.1%).

### Proportion of MRMP Infections by Variant Type

We performed subgroup analysis for variant types associated with MRMP infection (9745 samples in 131 data sets). Among the variants associated with resistance to macrolides in *M pneumoniae*, A2063G (96.8%; 95% CI, 95.8%-97.7%) was most commonly identified, followed by A2064G (4.8%; 95% CI, 3.5%-6.7%) (Table 1; eTable 5 and eTable 6 in the Supplement). In the Western Pacific region, the proportion of MRMP infections associated with A2063G showed an increasing trend with time, whereas the proportion of MRMP infections associated with A2064G showed a decreasing trend with time (Figure 3B and C; eTable 5 in the Supplement).

### Proportion of MRMP Infection by Age Group and RTI Type

When the study populations were classified by age group (children vs adults vs both children and adults), the proportion of MRMP infections was highest in studies that comprised only children (37.0%; 95% CI, 29.8%-46.1%), followed by those including only adults (15.9%; 95% CI, 6.4%-39.7%) and those comprising both children and adults (16.7%; 95% CI, 10.1%-27.6%) (Table 1). When the obtained samples were classified by type of RTI, the proportion of MRMP infections was higher in studies that included samples obtained from patients with CAP (38.8%; 95% CI, 30.3%-49.6%), followed by those that included samples obtained from patients with RTI (23.0%; 95% CI, 16.4%-32.4%).

Because MRMP infection has presented in children with CAP with aspects of refractory *M pneumoniae* pneumonia, we evaluated the proportion of MRMP infection according to the combination of age group and type of RTI (eTable 7 in the Supplement). The proportion of MRMP infections was highest in studies that included samples from children with CAP (43.9%; 95% CI, 34.2%-56.4%), followed by those that included samples from children with RTI (41.1%; 95% CI, 29.0%-58.2%), those that included samples from adults with CAP (12.4%; 95% CI, 2.6%-59.0%), and those that included samples from adults with RTI (11.2%; 95% CI, 2.6%-48.0%).

## Discussion

This systematic review and meta-analysis presents a considerable increase in global trends in the proportion of MRMP infections; specifically, we found that the overall proportion of MRMP infections increased from 18.2% in 2000 to 41.0% in 2010 to 76.5% in 2019. Geographical variations in the proportion of MRMP infections were apparent; the proportion of MRMP infections was highest in the Western Pacific region (53.4%), with a significantly increasing trend over time. Among the variant types associated with MRMP infections, A2063G was the most common variant (96.8%). Furthermore, the proportion of MRMP infections associated with the A2063G variant showed an increasing trend over time, whereas the proportion of MRMP infections associated with A2064G showed a decreasing trend in the Western Pacific region. The global proportion of MRMP infections was higher in samples from children than those from adults as well as in samples from patients with CAP than those with RTI.

There was only 1 systematic review on the proportion of MRMP infections, and that review focused solely on the European area.^29^ No studies have shown the global proportion of MRMP infections with time trends. To our knowledge, this study is the first systematic review and meta-analysis to investigate the global proportion of MRMP infections based on available data published from inception to 2021. The present study could serve as an important reference to describe the global and regional proportion of MRMP infections over time with differences in age groups and types of RTI.

The rationale behind the increasing patterns of MRMP infections has not been fully identified. A possible reason for this increase might be associated with the overuse of antibiotics, especially macrolides. A recently published study^30^ showed that a decreasing macrolide prescription rate was associated with a decreasing proportion of MRMP infections in Japan. In addition, the changing pattern over time of p1 genotypes in *M pneumoniae* might be temporally associated with the epidemiology of MRMP infections. To decrease the proportion of MRMP infections and develop appropriate therapeutics for MRMP infections, identification of the causes of the increasing trend in the proportion of MRMP infections is necessary. The results of the present systematic review and meta-analysis might play an important role by reporting timely and highly relevant baseline information on the proportion of MRMP infections over time, by region, and by variant type.

We found that the proportion of MRMP infections varied according to age group and type of RTI. The higher proportion of MRMP infections among children than adults might be associated with the fact that *M pneumoniae* is one of the most common causes of CAP, especially in children, and *M pneumoniae* can be more easily spread in group settings, such as schools.^1^ The higher proportion of MRMP infections among patients with CAP than among those with RTI might be partially associated with the higher use of medical facilities by patients with CAP than by those with RTI owing to the more severe clinical courses seen among patients with CAP, especially those with MRMP infection. The results of our study suggest that, when assessing the likelihood of MRMP infection in patients with *M pneumoniae* infection, considering patients’ RTI type and age may potentially lead to better clinical outcomes.

The proportion of MRMP infections associated with variant types varied by country and over time. We found A2063G to be the most common variant associated with MRMP infections. Although the proportion of MRMP infections associated with the A2064G variant is low, our study found that some countries, including Cuba, Germany, Italy, and Switzerland, have a relatively high proportion of MRMP infections (>30%) associated with this variant.^31,32,33,34,35^ In addition, rare but diverse types of variants associated with MRMP infection have been reported.^22,30,36^ Close monitoring for the advent of new variants and for changes in variant type associated with MRMP infections is required to control the spread of MRMP infection and to prevent treatment failure in *M pneumoniae* cases. In addition, attention to the variant types of MRMP infection according to geographical region is necessary for better prevention strategies to decrease the disease burden of MRMP infection due to regional differences in the variant types as well as the proportion of MRMP infections.

### Limitations

Our study has several limitations. First, there were large variations in the number of studies on the proportion of MRMP infections according to WHO regions and countries. We found that 67.3% of studies (103 of 153) were performed in the Western Pacific region, with China (n = 39) and Japan (n = 41) being the most common countries. These findings limit the generalization of the results. In general, more studies are likely to be performed where the prevalence of the disease is high. The results of the present meta-analysis can help researchers and clinicians better understand and identify the global and geographical proportion of MRMP infections. Based on the results of the meta-analysis, the trend in the proportion of MRMP infections needs to be distinguished by geographical region, although the proportion of MRMP infections is generally increasing. Second, the duration and timing of the studies varied, and the study periods partially overlapped in each study. Therefore, there might be gaps between the timing of tests and the estimation of MRMP infections each year. Third, substantial heterogeneities were present in the aspects of study population, age, type of RTI, severity of *M pneumoniae* infection, timing of sample collection, and variant type among the included studies. Nevertheless, we combined the studies in our meta-analysis because most cases of *M pneumoniae* infection are considered to be community-acquired infections, especially CAP in children; therefore, the results of the present meta-analysis can be generalized.

In the present study, we did not consider whether specimens were obtained from oropharyngeal swabs or nasopharyngeal swabs because only a few studies included such information, although there might be differences in sensitivity and specificity according to specimen type.^37^ Although culture is the criterion standard for detecting *M pneumoniae*, culture methods are not routinely used in clinical practice. Polymerase chain reaction methods have been widely used because of their high sensitivity, and PCR is widely used as a reference method to identify the presence of *M pneumoniae*. Therefore, we included studies using PCR for the diagnosis of *M pneumoniae* and the detection of MRMP infection. In the funnel plots of the proportion of MRMP infections using study sample size and the Egger test for investigation of small study biases, we found bias associated with sample size in studies of the A2063G and A2064G variants associated with MRMP infection (eFigure in the Supplement). Although studies on the proportion of MRMP infections inevitably have different sample sizes, the results of the present systematic review and meta-analysis are important despite these biases.

## Conclusions

This systematic review and meta-analysis has shown the global trends in the proportion of MRMP infections in terms of temporal trends, regional variations, and variant types. The increasing trend of MRMP infections reflects the high and increasing proportion of MRMP infection in the Western Pacific region. The A2063G variant was the most common variant associated with MRMP infection, but the A2064G variant and rare but diverse types of variants were also identified. The proportion of MRMP infections varies according to age group and type of RTI, and careful consideration of these factors is thus required when assessing macrolide resistance of *M pneumoniae* in clinical practice. The results of the present study provide helpful information on the proportion of MRMP infections and may be used to overcome the disease burden of MRMP infections via the establishment of appropriate therapeutic strategies.

## Notes

Supplement. **eAppendix.** Database Search Strategy **eTable 1.** Quality Assessment Scale for Rating the Risk of Bias **eTable 2.** Within-Study Bias Assessment for the Included Studies **eTable 3.** Summary of the Included Studies **eTable 4.** Proportion of MRMP in Each Country **eTable 5.** The Proportion of A2063G and A2064G Mutations by Year of Testing According to World Health Organization Regions in Studies With Information on Mutation Types Associated With Macrolide Resistance of *M pneumoniae* **eTable 6.** Proportion of Each Mutation Type Associated With Macrolide Resistance of *M pneumoniae* in Each Country **eTable 7.** Proportion of Macrolide Resistant *M pneumoniae* According to the Combination of Age Groups and RTI Types **eFigure.** Funnel Plots for the Proportion of Macrolide Resistance of *M pneumoniae* Against Study Sample Sizes and the Egger Test for Investigation of the Small Study Biases
eReferences.
