# Supplementary material for: Global Trends in the Proportion of Macrolide-Resistant Mycoplasma pneumoniae Infections: A Systematic Review and Meta-analysis
Source: JAMA Netw Open. 2022 Jul 11;5(7):e2220949. doi: 10.1001/jamanetworkopen.2022.20949 (PMC9274321; doi:10.1001/jamanetworkopen.2022.20949)
Supplement: Supplement. — eAppendix. Database Search Strategy eTable 1. Quality Assessment Scale for Rating the Risk of Bias eTable 2. Within-Study Bias Assessment for the Included Studies eTable 3. Summary of the Included Studies eTable 4. Proportion of MRMP in Each Country eTable 5. The Proportion of A2063G and A2064G Mutations by Year of Testing According to World Health Organization Regions in Studies With Information on Mutation Types Associated With Macrolide Resistance of M pneumoniae eTable 6. Proportion of Each Mutation Type Associated With Macrolide Resistance of M pneumoniae in Each Country eTable 7. Proportion of Macrolide Resistant M pneumoniae According to the Combination of Age Groups and RTI Types eFigure. Funnel Plots for the Proportion of Macrolide Resistance of M pneumoniae Against Study Sample Sizes and the Egger Test for Investigation of the Small Study Biases eReferences. [file jamanetwopen-e2220949-s001.pdf]

Supplementary Online Content

Kim K, Jung S, Kim M, Park S, Yang HJ, Lee E. Global trends in the proportion of macrolide-resistant *Mycoplasma pneumoniae* infections: a systematic review and meta-analysis. *JAMA Netw Open.* 2022;5(7):e2220949.
doi:10.1001/jamanetworkopen.2022.20949

eAppendix. Database Search Strategy
eTable 1. Quality Assessment Scale for Rating the Risk of Bias
eTable 2. Within-Study Bias Assessment for the Included Studies
eTable 3. Summary of the Included Studies
eTable 4. Proportion of MRMP in Each Country
eTable 5. The Proportion of A2063G and A2064G Mutations by Year of Testing According to World Health Organization Regions in Studies With Information on Mutation Types Associated With Macrolide Resistance of *M pneumoniae*
eTable 6. Proportion of Each Mutation Type Associated With Macrolide Resistance of *M. pneumoniae* in Each Country
eTable 7. Proportion of Macrolide Resistant *M pneumoniae* According to the Combination of Age Groups and RTI Types
eFigure. Funnel Plots for the Proportion of Macrolide Resistance of *M pneumoniae* Against Study Sample Sizes and the Egger Test for Investigation of the Small Study Biases
eReferences.

This supplementary material has been provided by the authors to give readers additional information about their work.

© 2022 Kim K et al. *JAMA Network Open.*

eAppendix. Database Search Strategy

PUBMED

‘mycoplasma pneumoniae’ OR mycoplasma pneumonia

AND resistance OR resistant OR macrolide

Filters: Humans; Field: MeSH Major Topic

EMBASE

‘mycoplasma pneumoniae’.af.

AND ‘resistant’.af. OR ‘resistance’.af. OR ‘macrolide’.af.

Cochrane

‘mycoplasma pneumoniae’.ab.ti.

‘macrolide’.ab.ti.

AND ‘resistance’:ab.ti OR ‘resistant’:ab.ti’ OR ‘macrolide’:ab.ti

© 2022 Kim K et al. *JAMA Network Open.*

eTable 1. Quality Assessment Scale for Rating the Risk of Bias

Bias type	Low risk (score=2)	Moderate risk (score=1)	High risk (score=3)
Sample population	1) Sample from the general population, not a select group<br>2) Consecutive unselected population<br>3) Rationale for case and control selection explained	1) Sample selected from large population but selection criteria not defined<br>2) Sample selection ambiguous but may be representative<br>3) Rationale for cases and controls not explained<br>4) Eligibility criteria not explained<br>5) Analysis to adjust for sampling strategy bias	1) Highly select population making it difficult to generalize finding<br>2) Sample selection ambiguous and sample unlikely to be representative
Sample size	1) Sample size calculation performed and adequate	1) Sample size calculation performed and reasons for not meeting sample size given<br>2) Sample size calculation not performed but all eligible persons studied	1) Sample size estimation unclear or only subsample studied
Participation rate	1) High response rate (>85%)	1) Moderate response rate (70-85%)	1) Low response rate (<70%)<br>2) Response rate not reported
Outcome assessment	1) Diagnosis using consistent criteria and direct examination	1) Assessment from administrative database or register<br>2) Assessment from hospital record or interviewer	1) Assessment from non-validated data or generic estimate from the overall population
Analytical methods to control for bias	1) Analysis appropriate for the type of sample (subgroup analysis/regression etc.)	1) Analysis does not account for common adjustment	1) Data confusing

© 2022 Kim K et al. *JAMA Network Open.*

eTable 2. Within-Study Bias Assessment for the Included Studies

Author Year	Sample population	Sample size	Participation	Outcome assessment	Analytical methods	Total score
CDC 2010[1]	2	1	2	1	2	8
Akaike H, 2012[2]	2	1	2	1	2	8
Akashi Y, 2018[3]	2	1	2	1	2	8
Ando M, 2018[4]	1	1	1	1	2	6
Averbuch D, 2011[5]	1	1	1	1	1	5
BAO Fang, 2013[6]	1	1	1	2	1	6
Beeton ML, 2020[7]	2	1	2	2	2	9
Big Mohammadi H, 2020[8]	1	1	1	1	1	5
Brown RJ, 2015[9]	1	1	2	2	1	7
Cao B, 2010[10]	2	1	2	2	1	8
Cardinale F, 2013[11]	1	1	2	2	1	7
Chalker VJ, 2012[12]	1	1	2	2	1	7
Chalker V, 2011[13]	2	1	2	2	1	8
Chalker V, 2012[14]	2	1	2	2	1	8
Chang CH, 2021[15]	1	1	2	2	1	7
Chen Y, 2018[16]	1	1	2	2	1	7
Cheong KN, 2016[17]	1	1	2	2	1	7
Chironna M, 2011[18]	1	1	2	2	1	7
Choi JH, 2019[19]	1	1	2	2	1	7
Copete AR, 2018[20]	2	1	2	2	1	8
Deng H, 2018[21]	2	1	2	2	1	8
Diaz MH, 2015[22]	2	1	2	2	1	8
Diaz MH, 2015[23]	1	1	2	2	1	7
Domthong P, 2016[24]	2	1	2	2	1	8
Domthong P, 2014[25]	1	1	2	2	1	7
Dong XP, 2013[26]	1	1	2	2	1	7
Dou HW, 2020[27]	1	1	2	2	1	7
Dumke R, 2013[28]	2	1	2	2	1	8
Dumke R, 2010[29]	2	1	2	2	1	8
Dumke R, 2019[30]	2	1	2	2	1	8
Eshaghi A, 2013[31]	1	1	2	2	1	7
Ferguson GD, 2013[32]	1	1	1	1	1	5
Goh A, 2014[33]	1	1	2	2	1	7
Gullsby K, 2016[34]	2	2	2	2	1	9
Gullsby K, 2019[35]	2	1	1	1	1	6
Guo D, 2019[36]	2	1	1	1	1	6
Guo DX, 2019[37]	2	1	2	1	1	7
Han HY, 2021[38]	2	1	2	1	1	7
Ho PL, 2015[39]	2	1	2	2	1	8
Hong KB, 2013[40]	1	1	2	2	1	7
Hung HM, 2021[41]	2	1	2	2	1	8
Ishiguro N, 2016[42]	1	1	2	2	1	7
Ishiguro N, 2017[43]	2	1	2	2	1	8
Ishiguro N, 2021[44]	1	1	2	2	1	7
Ishimaru N, 2021[45]	2	1	2	2	1	8
Katsukawa C, 2019[46]	1	1	2	2	1	7
Katsushima Y, 2015[47]	1	1	2	2	1	7
Kawai Y, 2012[48]	1	1	2	2	1	7
Kawai Y, 2013[49]	2	1	1	2	2	8
Kawai Y, 2013[50]	2	1	1	2	2	8
Kawakami N, 2021[51]	1	1	1	2	2	7
Kenri T, 2020[52]	2	1	1	2	2	8

© 2022 Kim K et al. *JAMA Network Open.*

Kim JH, 2017[53]	2	1	1	2	2	8
Kim MC, 2018[54]	1	1	1	2	2	7
Kim YJ, 2017[55]	1	1	1	2	2	7
Kogoj R, 2018[56]	1	1	1	2	2	7
Koike C, 2011[57]	1	1	1	2	2	7
Komatsu H, 2014[58]	1	1	1	2	2	7
Kurkela S, 2019[59]	2	1	1	2	2	8
Lanata M, 2021[60]	1	1	1	2	2	7
Leal S, 2020[61]	2	1	1	2	2	8
Lee E, 2017[62]	1	1	1	2	2	7
Lee H, 2021[63]	2	1	1	2	2	8
Lee JK, 2021[64]	2	1	1	2	2	8
Lee JK, 2018[65]	2	1	1	2	2	8
Li SL, 2012[66]	1	1	1	2	2	7
Lin C, 2010[67]	1	1	1	2	2	7
Liu X, 2014[68]	1	1	1	2	2	7
Liu Y, 2009[69]	1	1	1	2	2	7
Liu Y, 2010[70]	1	1	1	2	2	7
Liu Y, 2014[71]	1	1	1	2	2	7
Loconsole D, 2019[72]	2	1	1	2	2	8
Loo LH, 2017[73]	1	1	1	2	2	7
Lu C, 2020[74]	1	1	1	2	2	7
Lung D, 2013[75]	1	1	1	2	2	7
Ma Z, 2014[76]	1	1	1	2	2	7
Matsubara K, 2009[77]	1	1	1	2	2	7
Matsuda K, 2013[78]	1	1	1	2	2	7
Matsuoka M, 2004[79]	1	1	1	2	2	7
Meyer Sauteur, 2014[80]	1	1	1	2	2	7
Meyer Sauteur, 2021[81]	1	1	1	2	2	7
Miyashita N, 2013[82]	2	1	1	2	2	8
Miyashita N, 2011[83]	1	1	1	2	2	7
Miyashita N, 2012[84]	2	1	1	2	2	8
Miyashita N, 2010[85]	2	1	1	2	2	8
Miyashita, 2009[86]	1	1	1	2	2	7
Miyata, 2013[87]	1	1	1	2	2	7
Morimoto K, 2017[88]	2	1	1	2	2	8
Morinaga Y, 2020[89]	1	1	1	2	2	7
Morozumi M, 2013[90]	1	1	1	2	2	7
Morozumi M, 2005[91]	1	1	1	2	2	7
Morozumi M, 2008[92]	1	1	1	2	2	7
Morozumi M, 2020[93]	1	1	1	2	2	7
Muto T, 2021[94]	1	1	1	2	2	7
Nagita A, 2021[95]	1	1	1	2	2	7
Nakamura Y, 2021[96]	2	1	1	2	2	8
Nummi M, 2015[97]	1	1	1	2	2	7
Okada T, 2012[98]	2	1	1	2	2	8
Ouchi K, 2019[99]	2	1	1	2	2	8
Pereyre S, 2012[100]	0	1	2	2	2	7
Pereyre S, 2012[101]	0	1	2	2	2	7
Pereyre S, 2013[102]	2	1	2	2	2	9
Peuchant O, 2009[103]	1	1	0	2	2	6
Pouladi I, 2020[104]	0	0	2	2	2	6
Qu K, 2019[105]	2	1	2	2	2	9
Qu J, 2013[106]	2	1	2	2	2	9
Rivaya B, 2020[107]	2	1	2	2	2	9

© 2022 Kim K et al. *JAMA Network Open.*

Rodriguez N, 2019[108]	0	1	0	2	2	5
Smith, 2016[109]	0	1	0	2	2	5
Spuesens E, 2012[110]	2	1	1	2	2	8
Dumke R, 2009[111]	0	1	2	2	2	7
Sun H, 2017[112]	0	1	0	2	2	5
Suzuki S, 2018[113]	2	1	0	2	2	5
Suzuki Y, 2013[114]	0	1	0	2	2	5
Suzuki Y, 2017[115]	0	1	0	2	2	5
Tanaka T, 2017[116]	2	1	0	2	2	7
Uh Y, 2013[117]	0	1	0	2	2	5
Uldum S, 2012[118]	2	1	1	2	2	8
Voronina E, 2020[119]	0	1	0	2	2	5
Wagner K, 2019[120]	2	1	2	2	2	9
Waites K, 2019[121]	2	1	2	2	2	9
Waller J, 2014[122]	0	1	0	2	2	5
Wang Y, 2012[123]	0	1	2	2	2	7
Wang Y, 2021[124]	2	1	2	2	2	9
Wang Y, 2016[125]	0	1	2	2	2	7
Yin Y, 2017[126]	2	1	2	2	0	7
Whistler T, 2017[127]	2	1	0	2	2	7
Wu H, 2013[128]	2	1	2	2	2	9
Wu P, 2013[129]	2	1	2	2	2	9
Xiao L, 2020[130]	1	1	1	2	2	7
Xin D, 2008[131]	0	1	0	2	2	5
Xin D, 2009[132]	1	1	2	2	2	8
Xu C, 2021[133]	2	1	2	2	2	9
Xue G, 2018[134]	1	1	2	2	2	8
Xue G, 2014[135]	0	1	2	2	2	7
Yamada M, 2012[136]	1	1	2	2	2	8
Yan C, 2015[137]	0	1	0	2	2	5
Yan C, 2020[138]	2	1	2	2	2	9
Yin Y, 2017[139]	2	1	2	2	2	9
Yoo S, 2012[140]	0	1	2	2	2	7
Yu H, 2018[141]	2	1	2	2	2	9
Zhang W, 2019[142]	1	1	2	2	2	7
Zhao F, 2013[143]	2	1	2	2	2	9
Zhao F, 2019[144]	2	1	2	2	2	9
Zhao F, 2019[145]	0	1	0	2	2	5
Zhao H, 2014[146]	1	1	0	2	2	6
Zheng X, 2015[147]	0	1	0	2	2	5
Zhou Y, 2020[148]	2	1	2	2	2	9
Zhou Y, 2014[149]	2	1	2	2	2	9
Zhu M, 2020[150]	1	1	1	2	2	7

© 2022 Kim K et al. *JAMA Network Open.*

eTable 3. Summary of the Included Studies

Articles	Design	Country	Subject characteristics	Outcomes; RTI or CAP	Year	Age group	Total number of MP infection cases	Male (%)	A2063G, n	A2064G, n	Other mutation types (n)	MRMP, n (%)
CDC 2010[1]	Retrospective	USA	Outpatient & ED	CAP	2000	Children & Adults	11	NA	NA	NA	NA	2/11 (18.2)
Akaike H, 2012[2]	Retrospective	Japan	NA	RTI	2009-2011	Children	190	115 (60.5)	124	0	0	124/190 (65.2)
Akashi Y, 2018[3]	Retrospective	Japan	NA	RTI	2016-2017	Children & Adults	383	200 (52.2)	NA	NA	NA	221/383 (57.7)
Ando M, 2018[4]	Retrospective	Japan	NA	RTI	2002-2016	Children & Adults	417	NA	163	10	A2063C (10) C2617A, (1)	184/417 (44.1)
Averbuch D, 2011[5]	Retrospective	Israel	NA	RTI	2010-2010	Children & Adults	30	NA	7	0	A2063G (2)	9/30 (30.0)
BAO Fang, 2013[6]	Prospective	China	NA	CAP	2010-2011	Children	45	NA	NA	NA	NA	44/45 (98.0)
Beeton ML, 2020[7]	Retrospective	Israel	NA	RTI	2011-2016	Children & Adults	209	NA	NA	NA	NA	13/209 (1.5)
Big Mohammadi H, 2020[8]	Retrospective	Iran	NA	RTI	NA	Adults	100	48 (48)	0	0	2431G & AG2491A (1)	1/100 (1.0)
Brown RJ, 2015[9]	Retrospective	England	NA	CAP	2014-2015	Children & Adults	43	NA	0	0	A2054G (4)	4/43 (9.3)
Cao B, 2010[10]	Retrospective	China	NA	CAP	2008-2009	Children & Adults	67	NA	41	4	A2063T (1)	46/67 (68.7)
Cardinale F, 2013[11]	Retrospective	Italy	Inpatients	CAP	NA	Children	46	24 (52.2)	NA	NA	NA	8/46 (17.4)
Chalker VJ, 2012[12]	Retrospective	England	NA	NA	1995-2005, 2008-2011	NA	115	NA	1	0	0	1/115 (0.9)
Chalker V, 2011[13]	Retrospective	England	Outpatients	RTI	2010-2011	Children & Adults	12	NA	0	0	0	0/12 (0.0)
Chalker V, 2012[14]	Retrospective	England	NA	RTI	2011-2012	Children & Adults	12	NA	0	0	0	0/12 (0.0)

© 2022 Kim K et al. *JAMA Network Open.*

Chang CH, 2021[15]	Retrospectiv e	Taiwan	Inpatients	CAP	2016-2019	Children	81	35 (43.2)	45	0	0	45/81(54.3)
Chen Y, 2018[16]	Retrospectiv e	China	NA	CAP	2014-2016	Children	136	76 (55.9)	NA	NA	NA	81/136 (59.6)
Cheong KN, 2016[17]	Retrospectiv e	China	Inpatients	CAP	2011-2013	Children	93	42 (45.2)	NA	NA	NA	25/93 (26.9)
Chironna M, 2011[18]	Retrospectiv e	Italy	Inpatients	RTI	2010	Children	43	22 (51.2)	7	4	0	11/43 (25.6)
Choi JH, 2019[19]	Retrospectiv e	Korea	NA	RTI	2010-2016	Adults	70	34 (48.6)	2	0	0	2/70 (2.9)
Copete AR, 2018[20]	Retrospectiv e	Colom bia	Inpatients	CAP	2011-2012	Children	42	NA	0	0	0	0/42 (0.0)
Deng H, 2018[21]	Retrospectiv e	China	NA	CAP	2014-2015	Children	211	108 (51.2)	195	0	0	195/211 (92.4)
Diaz MH, 2015[22]	Retrospectiv e	USA	Inpatients	CAP	2010-2012	Children & Adults	216	124 (57.44)	6	1	0	7/216 (3.5)
Diaz MH, 2015[23]	Retrospectiv e	USA	NA	RTI	2006-2013	NA	176	NA	NA	NA	NA	19/176 (10.8)
Domthong P, 2016[24]	Retrospectiv e	Thailan d	NA	RTI	2012-2015	NA	116	NA	30	1	0	31/116 (26.7)
Domthong P, 2014[25]	Retrospectiv e, abstract	Thailan d	NA	RTI	2012-2013	NA	44	NA	16	0	0	16/44 (36.4)
Dong XP, 2013[26]	Retrospectiv e	China	Inpatients	CAP	2010	NA	53	NA	53	0	0	53/53 (100.0)
Dou HW, 2020[27]	Retrospectiv e	China	Outpatients & Inpatients	NA	2016	NA	214	NA	134	7	A2063G & A2064G (1)	142/214 (66.4)
Dumke R, 2013[28]	Retrospectiv e	Germa n	Outpatients & Inpatients	RTI	2009-2012	Children & Adults	84	NA	3	0	0	3/84 (3.6)
Dumke R, 2010[29]	Retrospectiv e	Germa n	NA	CAP	2003-2008, 1991-2009	Adults	266	NA	3	1	A2064C (1)	5/266 (1.9)
Dumke R, 2019[30]	Retrospectiv e	Germa n	NA	RTI	2016-2018	NA	166	NA	0	4	0	4/166 (2.4)
Eshaghi A, 2013[31]	Retrospectiv e	Canada	NA	RTI	2010-2012	Children & Adults	91	NA	6	1	Mixed with wide & 2063 &	11/91 (12.1)

© 2022 Kim K et al. *JAMA Network Open.*

											2064 (1), wild & 2063 (3)	
Ferguson GD, 2013[32]	Retrospective	UK	Outpatients & Inpatients	RTI	2010-2011	Children & Adults	32	NA	4	1	A2064C (1)	6/32 (18.8)
Goh A, 2014[33]	Retrospective	Singapore	NA	RTI	2012-2013	Children	28	NA	NA	NA	NA	8/28 (28.6)
Gullsby K, 2016[34]	Retrospective	Sweden	Outpatients & Inpatients	RTI	1996-2013	Children & Adults	548	269 (49.1)	0	0	0	0/548 (0.0)
Gullsby K, 2019[35]	Retrospective	Sweden	Outpatients & Inpatients	RTI	1996-2017	NA	578	NA	1	0	0	1/578 (0.2)
Guo D, 2019[36]	Retrospective	China	NA	RTI	2013-2015	Children,	164	NA	91	5	A2063G & A2064G (53)	149/164 (90.9)
Guo DX, 2019[37]	Retrospective	China	Inpatients	RTI	2014-2014	Children	341	NA	199	25	A2063G & A2064G (12)	236/341 (69.2)
Han HY, 2021[38]	Retrospective	Korea	Inpatients	CAP	2019-2020	Children	56	56	41	0	0	41/56 (73.2)
Ho PL, 2015[39]	Retrospective	Hong Kong	NA	RTI	2011-2014	Children & Adults	241	NA	84	0	NA	84/241 (34.9)
Hong KB, 2013[40]	Retrospective	Korea	NA	RTI	2000-2011	Children	225	NA	78	2	0	80/225 (35.6)
Hung HM, 2021[41]	prospective	Taiwan	NA	CAP	2017-2019	Children	226	102 (45.1)	144	2	A2063T (28)	174/226 (77.0)
Ishiguro N, 2016[42]	Retrospective	Japan	Outpatients & Inpatients	RTI	2012-2014	NA	95	NA	51	0	0	51/95 (53.7)
Ishiguro N, 2017[43]	prospective	Japan	Outpatients & Inpatients	CAP	2013-2015	Children	92	62 (67.4)	42	0	0	42/92 (45.7)

© 2022 Kim K et al. *JAMA Network Open.*

Ishiguro N, 2021[44]	Retrospective	Japan	NA	RTI	2013, 2014, 2017	Children	515	NA	153	2	C2617G (2)	157/515 (30.5)
Ishimaru N, 2021[45]	Retrospective	Japan	Outpatients & Inpatients	CAP	2016-2018	Adults	12	NA	NA	NA	NA	10/12 (83.3)
Katsukawa C, 2019[46]	Retrospective	Japan	Outpatients & Inpatients	RTI	2011-2017	Children	419	NA	209	0	A2063T (1)	210/419 (50.1)
Katsushima Y, 2015[47]	Retrospective	Japan	Outpatients	RTI	2012-2013	Children	27	16 (59.3)	23	0	C2617G (1)	24/27 (88.9)
Kawai Y, 2012[48]	Retrospective	Japan	Outpatients		2005-2010	Children	30	10 (33.3)	15	6	0	21/30 (70.0)
Kawai Y, 2013[49]	Prospective	Japan	NA	CAP	2005-2012	Children	188	106 (56.4)	134	6	A2063C (2) A2063T (8)	150/188 (80.0)
Kawai Y, 2013[50]	Prospective	Japan	NA	CAP	2008-2012	Children	769	297 (38.6)	538	3	A2063C (1) C2617G (1) A2063T(18)	561/769 (73.0)
Kawakami N, 2021[51]	Prospective	Japan	Outpatients	CAP	2015-2016	Children	151	72 (47.7)	83	2	0	85/151 (56.0)
Kenri T, 2020[52]	Retrospective	Japan	NA	NA	2006-2019	NA	554	NA	265	0	A2063C (1) A2063T (6) A2064C (5)	277/554 (50.0)
Kim JH, 2017[53]	Retrospective	Korea	NA	CAP	2011, 2015	Children	250	123 (49.2)	184	0	0	184/250 (74.0)
Kim MC, 2018[54]	Retrospective	Korea	NA	CAP	NA	Adults	70	34 (48.6)	2	0	0	2/70 (2.9)
Kim YJ, 2017[55]	Retrospective	Korea	NA	RTI	2010-2015	Children	107	44 (41.1)	11	0	0	11/107 (10.3)
Kogoj R, 2018[56]	Retrospective	Slovenia	NA	NA	2006-2015	NA	872	NA	0	0	A2058G (7)	7/872 (0.8)
Koike C, 2011[57]	Retrospective	Japan	NA	CAP	2006-2008	Children	16	9 (56.3)	12	0	0	12/16 (75.0)
Komatsu H, 2014[58]	Retrospective	Japan	NA	RTI	2010-2012	Children	33	16 (48.5)	30	1	0	31/33 (93.9)

© 2022 Kim K et al. *JAMA Network Open.*

Kurkela S, 2019[59]	Retrospective	Finland	NA	RTI	2017-2018	Children & Adult	12	NA	0	0	0	0/12 (0.0)
Lanata M, 2021[60]	Retrospective	US	NA	NA	2015-2019	Children	499	NA	11	3	0	14/499 (2.8)
Leal S, 2020[61]	Retrospective	US	NA	NA	2015-2019	NA	212	NA	NA	NA	NA	21/212 (9.9)
Lee E, 2017[62]	Retrospective	Korea	NA	CAP	2015	Children	94	37 (39.4)	82	0	0	82/94 (87.2)
Lee H, 2021[63]	Retrospective	Korea	NA	CAP	2014-2015, 2019-2020	Children	145	59 (40.7)	NA	NA	NA	59/145 (40.7)
Lee JK, 2021[64]	Retrospective	Korea	NA	CAP	2019-2020	NA	93	NA	NA	NA	NA	73/93 (78.5)
Lee JK, 2018[65]	Retrospective	Korea	NA	CAP	2000-2016	Children	146	NA	58	1	0	59/146 (40.4)
Li SL, 2012[66]	Retrospective	China	NA	CAP	2009-2010	Children	97	54 (55.7)	85	1	A2063T (2)	88/97 (90.7)
Lin C, 2010[67]	Retrospective	China	NA	CAP	2009	Children	64	38 (59.4)	57	0	A2063T (1)	58/64 (90.6)
Liu X, 2014[68]	Retrospective	China	NA	NA	2003-2007	NA	76	NA	64	5	A2063C (1)	70/76 (92.1)
Liu Y, 2009[69]	Retrospective	China	NA	RTI	2005-2008	Children	53	NA	44	0	0	44/53 (83.0)
Liu Y, 2010[70]	Retrospective	China	NA	RTI	2008-2009	Children	100	NA	88	1	A2063T (1)	90/100 (90.0)
Liu Y, 2014[71]	Retrospective	China	NA	RTI	2005-2008	Children	101	NA	81	1	0	82/101 (81.2)
Loconsole D, 2019[72]	Retrospective	Italy	NA	CAP	2013-2015	Adults	15	8 (53.3)	2	1	0	3/15 (20.0)
Loo LH, 2017[73]	Retrospective	Singapore	NA	NA	2013-2014	Children	200	NA	26	0	0	26/200 (13.0)
Lu C, 2020[74]	Retrospective	Taiwan	NA	CAP	2016	Children	180	NA	37	1	A2063T (5)	43/180 (23.9)
Lung D, 2013[75]	Retrospective	Hong Kong	NA	CAP	2010-2013	Children	48	NA	34	0	0	34/48 (70.8)
Ma Z, 2014[76]	Retrospective	China	NA	CAP	2010-2011	Children	57	39 (68.4)	36	0	0	36/57 (63.2)
Matsubara K, 2009[77]	Retrospective	Japan	NA	CAP	2002-2006	Children	94	NA	26	3	Unknown (1)	30/94 (31.9)

© 2022 Kim K et al. *JAMA Network Open.*

Matsuda K, 2013[78]	Prospective	Japan	NA	NA	2010-2011	Children	65	32 (49.2)	31	0	A2063T (27)	58/65 (89.2)
Matsuoka M, 2004[79]	Retrospective	Japan	NA	NA	2000-2003	Children	76	NA	10	1	A2063C (1) C2617G (1)	13/76 (17.1)
Meyer Sauteur, 2014[80]	Retrospective	Switzerland	NA	CAP	2011-2013	Children	50	NA	1	0	0	1/50 (2.0)
Meyer Sauteur, 2021[81]	Prospective	Switzerland	NA	CAP	2016-2017	Children	25	NA	NA	NA	NA	0/25 (0.0)
Miyashita N, 2013[82]	Retrospective	Japan	NA	CAP	2008-2012	Children & Adults	73	38 (52.1)	27	3	0	30/73 (41.1)
Miyashita N, 2011[83]	Retrospective	Japan	NA	CAP	2005-2010	Children	30	NA	NA	NA	NA	21/30 (70.0)
Miyashita N, 2012[84]	Retrospective	Japan	NA	CAP	2008-2011	Children & Adults	99	52 (52.5)	42	7	0	49/99 (49.5)
Miyashita N, 2010[85]	Retrospective	Japan	NA	CAP	2000-2009	Adults	84	NA	1	1	0	2/84 (2.4)
Miyashita, 2009[86]	Retrospective	Japan	NA	CAP	2005-2008	Children	21	NA	NA	NA	NA	14/21 (66.7)
Miyata, 2013[87]	Retrospective	Japan and US	NA	NA	NA	Children	Japan(21) US(18)	NA	Japan(16) US(1)	Japan (0) US (0)	Japan; A2063T (1) A2064C (1) US; 0	Japan 18/21 (85.7) US 1/18(5.6)
Morimoto K, 2017[88]	Retrospective	Japan	NA	CAP	2012-2015	Adults	55	NA	NA	NA	NA	32/55 (58.2)
Morinaga Y, 2020[89]	Retrospective	Japan	NA	NA	2016-2018	NA	249	NA	65	2	0	67/249 (26.9)
Morozumi M, 2013[90]	Prospective	Japan	NA	CAP	2008-2009	Children	54	NA	22	1	C2617A (1)	24/54 (44.4)
Morozumi M, 2005[91]	Prospective	Japan	NA	RTI	2002-2004	Children	195	NA	9	2	0	11/195 (5.6)
Morozumi M, 2008[92]	Prospective	Japan	NA	CAP	2002-2006	Children	380	NA	50	5	0	55/380 (14.5)

© 2022 Kim K et al. *JAMA Network Open.*

Morozumi M, 2020[93]	Prospective	Japan	NA	CAP	2018-2019	Children	53	NA	6	0	0	6/53 (11.3)
Muto T, 2021[94]	Retrospective	Japan	NA	NA	2018-2019	Children	21	10 (47.6)	NA	NA	NA	14/21 (66.7)
Nagita A, 2021[95]	Prospective	Japan	NA	CAP	2014-2017	Children	38	19 (50.0)	24	0	0	24/38 (63.1)
Nakamura Y, 2021[96]	Retrospective	Japan	NA	RTI	2008-2018	Children	1949	NA	1140	7	A2063C (3) C2617G (2) A2063T (30)	1182/1949 (60.6)
Nummi M, 2015[97]	Retrospective	Finland	NA	NA	NA	NA	42	NA	3	1	0	4/42 (9.5)
Okada T, 2012[98]	Prospective	Japan	NA	CAP	2011	Children	202	106 (52.5)	160	4	A2063C (1) A2063T (11)	176/202 (87.1)
Ouchi K, 2019[99]	NA	Japan	NA	NA	2008-2017	Children	1702	NA	NA	NA	NA	1180/1702 (69.3)
Pereyre S, 2012[100]	Retrospective	France, Israel	NA	RTI	2007-2010	Children & Adults	France (29) Israel (41)	NA	France (0) Israel (9)	France (1) Israel (0)	France (0) Israel (0)	France 1/29 (3.4) Israel 9/41 (22.0)
Pereyre S, 2012[101]	Retrospective	France	NA	RTI	2011	Children	6	NA	0	0	0	0/6 (0.0)
Pereyre S, 2013[102]	Retrospective	France	NA	NA	2000-2012	Children	72	NA	0	0	A2058G (4) A2059G (1) A2062G (1)	6/72 (8.3)
Peuchant O, 2009[103]	Retrospective	France	NA	NA	1998-2008	Children & Adults	67	NA	0	0	A2058G (3) A2059G (1) C2611G	5/67 (7.5)

© 2022 Kim K et al. *JAMA Network Open.*

											(1)	
Pouladi I, 2020[104]	Prospective	Iran	NA	RTI	2018	Adults	17	NA	0	0	0	0/17 (0.0)
Qu K, 2019[105]	Prospective	China	NA	CAP	2010-2012	Children & Adults	205	95 (46.3)	181	0	0	181/205 (88.3)
Qu J, 2013[106]	Prospective	China	NA	CAP	2010-2012	Children & Adults	136	61 (44.9)	114	0		114/136 (83.8)
Rivaya B, 2020[107]	Prospective	Spain	3 NA	CAP	2013-2017	Children	127	NA	7	2	A2063T & C2617A (1)	10/127 (7.8)
Rodriguez N, 2019[108]	Retrospective	Cuba	NA	CAP	2012, 2017	Children & Adults	27	NA	3	2	0	5/27 (18.5)
Smith, 2016[109]	Retrospective	Australia	NA	NA	1998-2014	Children & Adults	123	NA	0	0	0	0/123 (0.0)
Spuesens E, 2012[110]	Prospective	Netherlands	NA	RTI	1997-2008	Children & Adults	96	41 (42.7)	0	0	0	0/96 (0.0)
Dumke R, 2009[111]	Prospective	Germany	NA	CAP	1991-2009	Adults	266	NA	2	1	A2063C (1) A2063G (1)	5/266 (1.9)
Sun H, 2017[112]	Retrospective	China	NA	NA	2003-2007, 2014-2015	Children	149	NA	131	0	G2611T & T2613C (1)	132/149 (88.6)
Suzuki S, 2018[113]	Prospective	Japan	NA	RTI	2016-2017	Children	25	NA	7	0	0	7/25 (28.0)
Suzuki Y, 2013[114]	Retrospective	Japan	NA	RTI	2009-2010	Children	47	NA	0	0	A2063T (39)	39/47 (83.0)
Suzuki Y, 2017[115]	Retrospective	Japan	NA	NA	2004-2014	Children	347	NA	129	0	0	129/347 (37.2)
Tanaka T, 2017[116]	Prospective	Japan	NA	RTI	2008-2015	Children	1448	818 (56.5)	973	6	A2063C (3) A2063T (31) C2617G (2) C2617T (1)	1016/1448 (70.2)
Uh Y,	Prospective	Korea	NA	RTI	2010	Children	17	7 (41.2)	3	0	0	3/17 (17.6)

© 2022 Kim K et al. *JAMA Network Open.*

2013[117]												
Uldum S, 2012[118]	Retrospective	Denmark	NA	NA	2010-2011	NA	248	NA	NA	NA	NA	5/248 (2.0)
Voronina E, 2020[119]	Prospective	Russia	NA	RTI	2015-2018	NA	99	NA	7	0	0	7/99 (7.1)
Wagner K, 2019[120]	Retrospective	Switzerland	NA	RTI	2014-2017	NA	163	NA	9	5	A2063C (1)	15/163 (9.0)
Waites K, 2019[121]	Retrospective	USA	NA	RTI	2015-2018	Children	360	204 (56.7)	22	4	A2063T & A2063G (1)	27/360 (7.5)
Waller J, 2014[122]	Retrospective	USA	NA	CAP	2012-2012	Adults	21	NA	0	0	0	0/21(0.0)
Wang Y, 2012[123]	Retrospective	China	NA	RTI	2011	Children	15	12/15 (80.0)	0	0	2063 or 2617 mutation (12)	12/15 (80.0)
Wang Y, 2021[124]	Retrospective	China	NA	CAP	2016-2019	Children	1524	824 (54.1)	NA	NA	NA	1386/1524 (90.9)
Wang Y, 2016[125]	Prospective	China	NA	RTI	2014	Children	25	10 (40.0)	18	0	0	18/25 (72.0)
Yin Y, 2017[126]	Prospective	China	NA	CAP	2010-2012	Adults	75	NA	56	0	0	56/75 (74.7)
Whistler T, 2017[127]	Retrospective	Thailand	NA	RTI	2009-2012	Children & Adults	141	NA	0	0	0	0/141 (0.0)
Wu H, 2013[128]	Retrospective	Taiwan	NA	CAP	2011	Children	73	33 (45.2)	9	0	0	9/73 (12.3)
Wu P, 2013[129]	Prospective	Taiwan	NA	CAP	2010-2011	Children	60	NA	14	0	0	14/60 (23.3)
Xiao L, 2020[130]	Retrospective	USA	NA	RTI	2012-2014	Children	446	NA	32	NA	Unknown (5)	37/446 (8.3)
Xin D, 2008[131]	Retrospective	China	NA	NA	2004-2005	NA	64	NA	35	2	A2063C (1)	38/64 (59.4)
Xin D, 2009[132]	Retrospective	China	NA	RTI	2003-2006	Children	50	NA	40	5	A2063C (1)	46/50 (92.0)
Xu C, 2021[133]	Prospective	China	NA	CAP	2014-2016	Children	276	137 (49.6)	253	0	A2063G and G	255/276 (92.4)

© 2022 Kim K et al. *JAMA Network Open.*

											insertion between 2586 and 2587 (1) A2063G, G2601C, T insertion between 2589 and 2590, and T insertion between 2612 and 2613 (1)	
Xue G, 2018[134]	Retrospective	China	NA	RTI	2016	Children	213	NA	137	0	A2063G & A2065C (1), A2063G & wild type (3), A2064G & wild type (1)	142/213 (76.3)
Xue G, 2014[135]	Retrospective	Australia , China	NA	NA	2008-2012	Children & Adults	Australia (30) , China (83)	NA	Australia (0) China (68)	Australia (1) China (0)	Australia (0) China A2063C (3)	Australia 1/30 (3.3) , China 71/83 (85.5)
Yamada M, 2012[136]	Retrospective	Japan	NA	NA	2007-2010	Children	49	NA	2	0	wild type (2)	4/49 (8.2)
Yan C, 2015[137]	Retrospective	USA	NA	NA	2012-2014	Children	59	NA	7	0	0	7/59 (11.9)
Yan C, 2020[138]	Prospective	China	NA	CAP	2014-2015	Children & Adults	247	NA	209	5	A2063T (2) C2617T (1)	217/247 (87.9)
Yin Y, 2017[139]	Prospective	China	NA	CAP	2010-2012	Adults	75	30 (40.0)	53	1	0	54/75 (77.1)
Yoo S,	Retrospective	Korea	NA	CAP	2011	Children &	91	39 (42.9)	27	0	0	27/91 (29.7)

© 2022 Kim K et al. *JAMA Network Open.*

2012[140]	e					Adults						
Yu H, 2018[141]	Retrospectiv e	China	NA	CAP	2015-2016	Adults	27	11 (40.7)	27	0	0	27/27 (100.0)
Zhang W, 2019[142]	Retrospectiv e	China	NA	RTI	2018	Children	19	NA	19	0	0	19/19 (100.0)
Zhao F, 2013[143]	Retrospectiv e	China	NA	RTI	2008-2012	Children & Adults	309	NA	272	7	A2063T (1)	280/309 (90.6)
Zhao F, 2019[144]	Retrospectiv e	China	NA	NA	2017-2018	Children	154	NA	121	2	0	123/154 (79.9)
Zhao F, 2019[145]	Retrospectiv e	China	NA	RTI	2014-2016	Children	81	33 (40.7)	52	1	0	53/81 (65.4)
Zhao H, 2014[146]	Retrospectiv e	China	NA	RTI	2007-2012	Children	129	NA	116	0	A2063G & T2611C (1)	117/129 (90.7)
Zheng X, 2015[147]	Retrospectiv e	USA	NA	NA	2012-2014	Children & Adults	91	NA	10	NA	Unknown (2)	12/91 (13.2)
Zhou Y, 2020[148]	Retrospectiv e	China	NA	CAP	2016-2019	Children	107	55 (51.4)	60	0	0	60/107 (56.1)
Zhou Y, 2014[149]	Retrospectiv e	China	NA	CAP	2009- 2010	Children	235	149 (63.4)	199	1	A2063T (6)	206/235 (87.7)
Zhu M, 2020[150]	Retrospectiv e	China	NA	RTI	2015-2018	Children	315	177 (56.2)	164	0	0	164/315 (52.1)

CAP, community-acquired pneumonia; MP, *M pneumoniae* ; MRMP, macrolide resistant *M pneumoniae*; n, number; NA, not applicable; RTI, respiratory tract infection.

© 2022 Kim K et al. *JAMA Network Open.*

eTable 4. Proportion of MRMP in Each Country

WHO regions	Country	Number of studies in each country	MRMP/total, n (%)	Summary estimate (95% CI)
AMR		14	163/2269 (7.2)	8.4% (6.1-11.6)
	Cuba	1	5/27 (18.5)	18.5% (8.4-40.9)
	Canada	1	11/91 (12.1)	12.1% (7.0-21.0)
	USA	11	147/2109 (7..0)	7.7% (5.4-11.0)
	Colombia	1	0/42 (0.0)	1.2% (0.1-18.5)
EMR		2	1/117 (0.9)	1.4% (0.3-7.0)
	Iran	2	1/117 (0.9)	1.4% (0.3-7.0)
EUR		31	144/4414 (3.3)	5.1% (3.3-8.0)
	Italy	3	22/104 (21.2)	21.7% (15.0-31.4)
	Israel	3	31/280 (11.1)	15.9% (6.2-41.1)
	Slovenia	1	7/872 (8.0)	0.8% (0.4-1.7)
	Spain	1	10/127 (7.9)	7.9% (4.3-14.3)
	Finland	2	4/54 (7.4)	8.7% (3.6-21.0)
	Russia	1	7/99 (7.1)	7.1% (3.5-14.4)
	France	4	12/174 (6.9)	7.4% (4.4-12.7)
	Switzerland	3	16/238 (6.7)	5.1% (1.5-16.7)
	UK	5	11/214 (5.1)	6.4% (2.1-19.4)
	German	4	18/782 (2.3)	2.4% (1.5-3.8)
	Denmark	1	5/248 (2.0)	2.0% (0.9-4.8)
	Sweden	2	1/1126 (0.1)	0.1% (0.0-0.7)
	Netherlands	1	0/96 (0.0)	0.5% (0.0-8.2)
SEAR		3	47/301 (15.6)	9.8% (0.8-100.0)
	Thailand	3	47/301 (15.6)	9.8% (0.8-100.0)
WPR		103	12634/20307 (62.2)	53.4% (47.4-60.3)
	China	39	5210/6385 (81.6)	79.5% (74.6-84.8)
	Japan	41	6363/11268 (56.5)	47.3% (38.9-57.5)
	Taiwan	5	285/620 (46.0)	32.4% (17.1-61.2)
	Korea	12	623/1364 (45.7)	30.0% (16.6-54.1)
	Hong Kong	2	118/289 (40.8)	49.7% (24.8-99.5)

© 2022 Kim K et al. *JAMA Network Open.*

	Singapore	2	34/228 (14.9)	18.6% (8.6-40.1)
	Australia	2	1/153 (0.7)	1.5% (0.2-11.1)
	Total	153	12989/27408 (47.0)	27.5% (22.5-33.5)

AMR, Region of the Americas; EMR, Eastern Mediterranean Region EUR, European Region; NA, not applicable; SEAR, South-East Asian Region; WPR, Western Pacific Region.

© 2022 Kim K et al. *JAMA Network Open.*

eTable 5. The Proportion of A2063G and A2064G Mutations by Year of Testing According to World Health Organization Regions in Studies With Information on Mutation Types Associated With Macrolide Resistance of *M pneumoniae*

Year of testing	Global proportion, %		Region of the Americas, %		European Region, %		Western Pacific Region, %	
	A2063G	A2064G	A2063G	A2064G	A2063G	A2064G	A2063G	A2064G
2001	76.9	7.7	NA	NA	NA	NA	76.9	7.7
2002	NA	NA	NA	NA	NA	NA	NA	NA
2003	56.3.	12.5	NA	NA	NA	NA	81.8	18.2
2004	94.0	5.4	NA	NA	NA	NA	94.0	5.4
2005	92.9	5.2	NA	NA	40.0	20.0	94.7	4.7
2006	94.7	0.8	NA	NA	0.0	0.0	99.2	0.8
2007	82.4	17.6	NA	NA	100.0	0.0	81.8	18.2
2008	92.4	3.7	NA	NA	90.0	10.0	92.5	3.5
2009	89.6	1.6	NA	NA	NA	NA	89.6	1.
2010	93.7	1.5	NA	NA	58.3	13.9	94.7	1.1
2011	95.4	0.8	66.7	11.1	NA	NA	95.7	0.7
2012	96.9	0.0	NA	NA	100.0	0.0	96.7	0.0
2013	96.6	0.6	96.1	0.0	NA	NA	96.7	0.6
2014	90.5	3.4	NA	NA	28.6	14.3	90.8	3.3
2015	97.3	1.8	NA	NA	61.5	26.9	99.0	0.6
2016	95.7	2.3	81.5	14.8	100.0	0.0	96.4	1.6
2017	95.5	4.5	78.6	21.4	0.0	100.0	97.6	2.4
2018	84.9	1.0	NA	NA	NA	NA	84.9	1.0
2019	100.0	0.0	NA	NA	NA	NA	100.0	0.0

MRMP, macrolide-resistant *M pneumoniae*; NA, not applicable.

© 2022 Kim K et al. *JAMA Network Open.*

eTable 6. Proportion of Each Mutation Type Associated With Macrolide Resistance of *M. pneumoniae* in Each Country

Countries	A2063G, n (%)	A2064G, n (%)	Other mutations, n (%)
Australia	0 (0.0%)	1 (100.0%)	0 (0.0%)
Canada	6 (54.5%)	1 (9.1%)	4 (36.4%)
China	3493 (95.1%)	73 (2.0%)	107 (2.9%)
Cuba	3 (60.0%)	2 (40.0%)	0 (0.0%)
Finland	3 (75.0%)	1 (25.0%)	0 (0.0%)
France	0 (0.0%)	1 (8.3%)	11 (91.7%)
Germany	8 (44.4%)	6 (33.3%)	4 (22.2%)
Hong Kong	118 (100.0%)	0 (0.0%)	0 (0.0%)
Iran	0 (0.0%)	0 (0.0%)	1 (100.0%)
Israel	16 (88.9%)	0 (0.0%)	2 (11.1%)
Italy	9 (64.3%)	5 (35.7%)	0 (0.0%)
Japan	4579 (94.1%)	75 (1.5%)	214 (4.4%)
Korea	488 (99.4%)	3 (0.6%)	0 (0.0%)
Russia	7 (100.0%)	0 (0.0%)	0 (0.0%)
Singapore	26 (100.0%)	0 (0.0%)	0 (0.0%)
Slovenia	0 (0.0%)	0 (0.0%)	7 (100.0%)
Spain	7 (63.6%)	2 (18.2%)	2 (18.2%)
Sweden	1 (100.0%)	0 (0.0%)	0 (0.0%)
Switzerland	10 (62.5%)	5 (31.2%)	1 (6.2%)
Taiwan	249 (87.4%)	3 (1.1%)	33 (11.6%)
Thailand	46 (97.9%)	1 (2.1%)	0 (0.0%)
UK	5 (45.5%)	1 (9.1%)	5 (45.5%)
USA	89 (89.0%)	8 (8.0%)	3 (3.0%)

© 2022 Kim K et al. *JAMA Network Open.*

eTable 7. Proportion of Macrolide Resistant *M pneumoniae* According to the Combination of Age Groups and RTI Types

Variable	No. of articles	Positive samples/total samples	Proportion (95% CI)	p value for χ^2		
				Q test	Egger test	Begg test
Children with CAP						
MRMP in studies with or without information on mutation types	39	4315/6606	43.9% (34.2-56.4)	<0.001	<0.001	<0.001
A2063G	30	2510/2642	96.1% (94.1-98.0)	<0.001	<0.001	0.1045
A2064G	24	45/2642	4.2% (2.4-7.6)	<0.001	0.0718	0.024
Other mutations	15	87/2642	4.7% (2.7-8.1)	<0.001	0.0654	0.255
MRMP in studies with information on mutation types	30	2642/6606	39.8% (29.2-54.2)	<0.001	<0.001	0.001
Children with RTI						
MRMP in studies with or without information on mutation types	29	2751/5814	41.1% (29.0-58.2)	<0.001	0.004	0.0055
A2063G	26	2588/2746	98.0% (96.6-99.4)	<0.001	0.0239	0.0051
A2064G	20	47/2746	2.5% (1.2-5.1)	<0.001	0.0103	0.0015
Other mutations	17	111/2746	3.8% (1.6-9.2)	<0.001	<0.001	0.6211
MRMP in studies with information on mutation types	29	2746/5814	40.8% (28.6-58.1)	<0.001	0.0039	0.0055
Adults with CAP						
MRMP in studies with or without information on mutation types	7	142/531	12.4% (2.6-59.0)	<0.001	0.0115	0.6523
A2063G	4	138/142	99.5% (97.6-100.0)	0.3209	0.1380	0.1742
A2064G	2	2/142	6.3% (0.6-65.0)	0.0746	NA	NA
Other mutations	2	2/142	7.6% (0.2-100.0)	0.0124	NA	NA
MRMP in studies with information on mutation types	5	142/531	20.3% (3.3-100.0)	<0.001	0.028	0.3272
Adults with RTI						
MRMP in studies with or without information on mutation types	6	1290/2423	11.2% (2.6-48.0)	<0.001	0.3114	0.3476
A2063G	4	1234/1288	96.6% (95.6-97.6)	0.055	0.2515	0.4969
A2064G	4	13/1288	5.2% (0.5-56.9)	<0.001	0.3422	0.4969
Other mutations	5	41/1288	11.3% (2.2-58.1)	<0.001	0.3756	0.6242
MRMP in studies with information on mutation types	6	1288/2423	10.7% (2.4-47.6)	<0.001	0.3172	0.3476

CAP, community-acquired pneumonia; CI, confidence interval; MRMP, macrolide resistant *M pneumoniae*; NA, not applicable; RTI, respiratory tract infection.

eFigure. Funnel Plots for the Proportion of Macrolide Resistance of *M pneumoniae* Against Study Sample Sizes and the Egger Test for Investigation of the Small Study Biases. (A) Studies for A2063G mutation associated with macrolide resistance of *M pneumoniae*. (B) Studies for A2064G mutation associated with macrolide resistance of *M pneumoniae*.

A

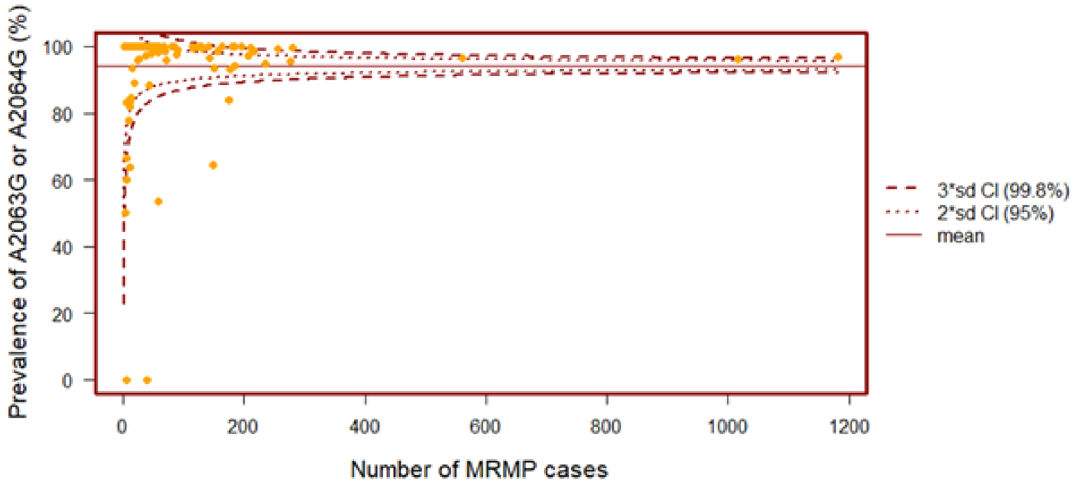

B

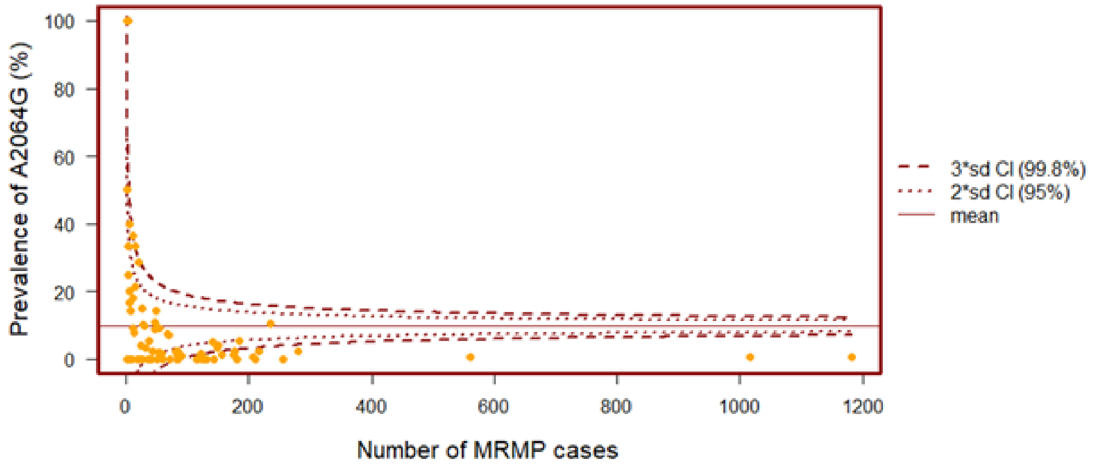

© 2022 Kim K et al. *JAMA Network Open.*

eReferences.

1. Mycoplasma pneumoniae respiratory illness - two rural counties, West Virginia, 2011. *MMWR Morb Mortal Wkly Rep*. Oct 19 2012;61(41):834-8.

2. Akaike H, Miyashita N, Kubo M, et al. In vitro activities of 11 antimicrobial agents against macrolide-resistant Mycoplasma pneumoniae isolates from pediatric patients: results from a multicenter surveillance study. *Jpn J Infect Dis*. 2012;65(6):535-8. doi:10.7883/yoken.65.535

3. Akashi Y, Hayashi D, Suzuki H, et al. Clinical features and seasonal variations in the prevalence of macrolide-resistant Mycoplasma pneumoniae. *J Gen Fam Med*. Nov 2018;19(6):191-197. doi:10.1002/jgf2.201

4. Ando M, Morozumi M, Adachi Y, Ubukata K, Iwata S. Multilocus sequence typing of Mycoplasma pneumoniae, Japan, 2002–2016. Article. *Emerging Infectious Diseases*. 2018;24(10):1898-1901. doi:10.3201/eid2410.171194

5. Averbuch D, Hidalgo-Grass C, Moses AE, Engelhard D, Nir-Paz R. Macrolide resistance in Mycoplasma pneumoniae, Israel, 2010. *Emerg Infect Dis*. Jun 2011;17(6):1079-82. doi:10.3201/eid/1706.101558

6. Bao F, Qu JX, Liu ZJ, Qin XG, Cao B. [The clinical characteristics, treatment and outcome of macrolide-resistant Mycoplasma pneumoniae pneumonia in children]. *Zhonghua Jie He He Hu Xi Za Zhi*. Oct 2013;36(10):756-61.

7. Beeton ML, Zhang XS, Uldum SA, et al. Mycoplasma pneumoniae infections, 11 countries in Europe and Israel, 2011 to 2016. *Euro Surveill*. Jan 2020;25(2)doi:10.2807/1560-7917.Es.2020.25.2.1900112

8. Big Mohammadi H, Pouladi I, Zolfaghari MR, Niakan M. The Prevalence of 23S rRNA Mutations in ML-Resistant M. pneumoniae Isolates to Clarithromycin in Patients with Respiratory Infections. *Rep Biochem Mol Biol*. Jul 2020;9(2):156-162. doi:10.29252/rbmb.9.2.156

9. Brown RJ, Macfarlane-Smith L, Phillips S, Chalker VJ. Detection of macrolide resistant Mycoplasma pneumoniae in England, September 2014 to September 2015. *Euro Surveill*. 2015;20(48):30078. doi:10.2807/1560-7917.Es.2015.20.48.30078

10. Cao B, Zhao CJ, Yin YD, et al. High prevalence of macrolide resistance in Mycoplasma pneumoniae isolates from adult and adolescent patients with respiratory tract infection in China. *Clin Infect Dis*. Jul 15 2010;51(2):189-94. doi:10.1086/653535

11. Cardinale F, Chironna M, Chinellato I, Principi N, Esposito S. Clinical relevance of Mycoplasma

© 2022 Kim K et al. *JAMA Network Open.*

pneumoniae macrolide resistance in children. *J Clin Microbiol*. Feb 2013;51(2):723-4. doi:10.1128/jcm.02840-12

12. Chalker VJ, Stocki T, Mentasti M, Harnden A, Wang K, Harrison TG. Macrolide resistant Mycoplasma pneumoniae in England and Wales. Conference Abstract. *Clinical Microbiology and Infection*. 2012;18:135. doi:10.1111/j.1469-0691.2012.03802.x

13. Chalker V, Stocki T, Mentasti M, Fleming D, Harrison T. Increased incidence of Mycoplasma pneumoniae infection in England and Wales in 2010: multiocus variable number tandem repeat analysis typing and macrolide susceptibility. *Euro Surveill*. May 12 2011;16(19)

14. Chalker V, Stocki T, Litt D, et al. Increased detection of Mycoplasma pneumoniae infection in children in England and Wales, October 2011 to January 2012. *Euro Surveill*. Feb 9 2012;17(6)

15. Chang CH, Tsai CK, Tsai TA, et al. Epidemiology and clinical manifestations of children with macrolide-resistant Mycoplasma pneumoniae pneumonia in Southern Taiwan. *Pediatr Neonatol*. Sep 2021;62(5):536-542. doi:10.1016/j.pedneo.2021.05.017

16. Chen Y, Tian WM, Chen Q, et al. [Clinical features and treatment of macrolide-resistant Mycoplasma pneumoniae pneumonia in children]. *Zhongguo Dang Dai Er Ke Za Zhi*. Aug 2018;20(8):629-634. doi:10.7499/j.issn.1008-8830.2018.08.006

17. Cheong KN, Chiu SS, Chan BW, To KK, Chan EL, Ho PL. Severe macrolide-resistant Mycoplasma pneumoniae pneumonia associated with macrolide failure. *J Microbiol Immunol Infect*. Feb 2016;49(1):127-30. doi:10.1016/j.jmii.2014.11.003

18. Chironna M, Sallustio A, Esposito S, et al. Emergence of macrolide-resistant strains during an outbreak of Mycoplasma pneumoniae infections in children. *J Antimicrob Chemother*. Apr 2011;66(4):734-7. doi:10.1093/jac/dkr003

19. Choi JH, Seong GM, Ko Y, Kim YR, Kim C. Prevalence and Clinical Features of Community-Acquired Pneumonia Caused by Macrolide-Resistant Mycoplasma pneumoniae Isolated from Adults in Jeju Island. *Microb Drug Resist*. May 2019;25(4):577-581. doi:10.1089/mdr.2018.0295

20. Copete AR, Aguilar YA, Rueda ZV, Vélez LA. Genotyping and macrolide resistance of Mycoplasma pneumoniae identified in children with community-acquired pneumonia in Medellín, Colombia. *Int J Infect Dis*. Jan 2018;66:113-120. doi:10.1016/j.ijid.2017.11.019

21. Deng H, Rui J, Zhao D, Liu F. Mycoplasma pneumoniae 23S rRNA A2063G mutation does not influence chest radiography features in children with pneumonia. *J Int Med Res*. Jan 2018;46(1):150-157.
© 2022 Kim K et al. *JAMA Network Open.*

doi:10.1177/0300060517716312

22. Diaz MH, Benitez AJ, Cross KE, et al. Molecular Detection and Characterization of Mycoplasma pneumoniae Among Patients Hospitalized With Community-Acquired Pneumonia in the United States. *Open Forum Infect Dis*. Sep 2015;2(3):ofv106. doi:10.1093/ofid/ofv106

23. Diaz MH, Benitez AJ, Winchell JM. Investigations of Mycoplasma pneumoniae infections in the United States: trends in molecular typing and macrolide resistance from 2006 to 2013. *J Clin Microbiol*. Jan 2015;53(1):124-30. doi:10.1128/jcm.02597-14

24. Domthong P, Domthong U, Hansiriphan P, et al. Macrolide-resistant mycoplasma pneumoniae in respiratory tract infection and its clinical relevance. Conference Abstract. *European Respiratory Journal*. 2016;48doi:10.1183/13993003.congress-2016.PA2614

25. Domthong P, Wattanathum A, Harnsiripan P, Tanyaharn S, Wutthichan V. Emerging macrolide-resistant mycoplasma pneumoniae in Thailand. Conference Abstract. *European Respiratory Journal*. 2014;44:P2493.

26. Dong XP, Dong YQ, Ma L, Zhang ZH, Jiang Y, Xin DL. Surveillance of drug-resistance in Mycoplasma pneumoniae and analysis of clinical features of Mycoplasma pneumoniae pneumonia in childhood. *Chin Med J (Engl)*. Nov 2013;126(22):4339.

27. Dou HW, Tian XJ, Xin L, et al. Mycoplasma pneumoniae Macrolide Resistance and MLVA Typing in Children in Beijing, China, in 2016: Is It Relevant? *Biomed Environ Sci*. Dec 20 2020;33(12):916-924. doi:10.3967/bes2020.125

28. Dumke R, Lück C, Jacobs E. Low rate of macrolide resistance in Mycoplasma pneumoniae strains in Germany between 2009 and 2012. *Antimicrob Agents Chemother*. Jul 2013;57(7):3460. doi:10.1128/aac.00706-13

29. Dumke R, von Baum H, Lück PC, Jacobs E. Occurrence of macrolide-resistant Mycoplasma pneumoniae strains in Germany. *Clin Microbiol Infect*. Jun 2010;16(6):613-6. doi:10.1111/j.1469-0691.2009.02968.x

30. Dumke R, Ziegler T. Long-Term Low Rate of Macrolide-Resistant Mycoplasma pneumoniae Strains in Germany. *Antimicrob Agents Chemother*. May 2019;63(5)doi:10.1128/aac.00455-19

31. Eshaghi A, Memari N, Tang P, et al. Macrolide-resistant Mycoplasma pneumoniae in humans, Ontario, Canada, 2010-2011. *Emerg Infect Dis*. 2013;19(9):1525-7. doi:10.3201/eid1909.121466

32. Ferguson GD, Gadsby NJ, Henderson SS, et al. Clinical outcomes and macrolide resistance in

Mycoplasma pneumoniae infection in Scotland, UK. *J Med Microbiol*. Dec 2013;62(Pt 12):1876-1882. doi:10.1099/jmm.0.066191-0

33. Goh A, Loo LH, Chan YH, et al. Macrolide-resistant mycoplasma pneumoniae-is it relevant? Conference Abstract. *Pediatric Pulmonology*. 2014;49:S67-S68. doi:10.1002/ppul.23068

34. Gullsby K, Bondeson K. No detection of macrolide-resistant Mycoplasma pneumoniae from Swedish patients, 1996-2013. *Infect Ecol Epidemiol*. 2016;6:31374. doi:10.3402/iee.v6.31374

35. Gullsby K, Olsen B, Bondeson K. Molecular Typing of Mycoplasma pneumoniae Strains in Sweden from 1996 to 2017 and the Emergence of a New P1 Cytadhesin Gene, Variant 2e. *J Clin Microbiol*. Jun 2019;57(6)doi:10.1128/jcm.00049-19

36. Guo D, Hu W, Xu B, et al. Allele-specific real-time PCR testing for minor macrolide-resistant Mycoplasma Pneumoniae. *BMC Infect Dis*. Jul 12 2019;19(1):616. doi:10.1186/s12879-019-4228-4

37. Guo DX, Hu WJ, Wei R, et al. Epidemiology and mechanism of drug resistance of Mycoplasma pneumoniae in Beijing, China: A multicenter study. *Bosn J Basic Med Sci*. Aug 20 2019;19(3):288-296. doi:10.17305/bjbms.2019.4053

38. Han HY, Park KC, Yang EA, Lee KY. Macrolide-Resistant and Macrolide-Sensitive Mycoplasma pneumoniae Pneumonia in Children Treated Using Early Corticosteroids. *J Clin Med*. Mar 22 2021;10(6)doi:10.3390/jcm10061309

39. Ho PL, Law PY, Chan BW, et al. Emergence of Macrolide-Resistant Mycoplasma pneumoniae in Hong Kong Is Linked to Increasing Macrolide Resistance in Multilocus Variable-Number Tandem-Repeat Analysis Type 4-5-7-2. *J Clin Microbiol*. Nov 2015;53(11):3560-4. doi:10.1128/jcm.01983-15

40. Hong KB, Choi EH, Lee HJ, et al. Macrolide resistance of Mycoplasma pneumoniae, South Korea, 2000-2011. *Emerg Infect Dis*. Aug 2013;19(8):1281-4. doi:10.3201/eid1908.121455

41. Hung HM, Chuang CH, Chen YY, et al. Clonal spread of macrolide-resistant Mycoplasma pneumoniae sequence type-3 and type-17 with recombination on non-P1 adhesin among children in Taiwan. *Clin Microbiol Infect*. Aug 2021;27(8):1169.e1-1169.e6. doi:10.1016/j.cmi.2020.09.035

42. Ishiguro N, Koseki N, Kaiho M, et al. Regional Differences in Prevalence of Macrolide Resistance among Pediatric Mycoplasma pneumoniae Infections in Hokkaido, Japan. *Jpn J Infect Dis*. May 20 2016;69(3):186-90. doi:10.7883/yoken.JJID.2015.054

43. Ishiguro N, Koseki N, Kaiho M, et al. Therapeutic efficacy of azithromycin, clarithromycin, minocycline and tosufloxacin against macrolide-resistant and macrolide-sensitive Mycoplasma pneumoniae

pneumonia in pediatric patients. *PLoS One*. 2017;12(3):e0173635. doi:10.1371/journal.pone.0173635

44. Ishiguro N, Sato R, Kikuta H, et al. P1 gene of Mycoplasma pneumoniae isolated from 2016 to 2019 and relationship between genotyping and macrolide resistance in Hokkaido, Japan. *J Med Microbiol*. Jun 2021;70(6)doi:10.1099/jmm.0.001365

45. Ishimaru N, Suzuki S, Shimokawa T, et al. Predicting Mycoplasma pneumoniae and Chlamydophila pneumoniae in community-acquired pneumonia (CAP) pneumonia: epidemiological study of respiratory tract infection using multiplex PCR assays. *Intern Emerg Med*. May 13 2021:1-9. doi:10.1007/s11739-021-02744-6

46. Katsukawa C, Kenri T, Shibayama K, Takahashi K. Genetic characterization of Mycoplasma pneumoniae isolated in Osaka between 2011 and 2017: Decreased detection rate of macrolide-resistance and increase of p1 gene type 2 lineage strains. *PLoS One*. 2019;14(1):e0209938. doi:10.1371/journal.pone.0209938

47. Katsushima Y, Katsushima F, Suzuki Y, et al. Characteristics of Mycoplasma pneumoniae infection identified on culture in a pediatric clinic. *Pediatr Int*. Apr 2015;57(2):247-52. doi:10.1111/ped.12513

48. Kawai Y, Miyashita N, Yamaguchi T, et al. Clinical efficacy of macrolide antibiotics against genetically determined macrolide-resistant Mycoplasma pneumoniae pneumonia in paediatric patients. *Respirology*. Feb 2012;17(2):354-62. doi:10.1111/j.1440-1843.2011.02102.x

49. Kawai Y, Miyashita N, Kubo M, et al. Therapeutic efficacy of macrolides, minocycline, and tosufloxacin against macrolide-resistant Mycoplasma pneumoniae pneumonia in pediatric patients. *Antimicrob Agents Chemother*. May 2013;57(5):2252-8. doi:10.1128/aac.00048-13

50. Kawai Y, Miyashita N, Kubo M, et al. Nationwide surveillance of macrolide-resistant Mycoplasma pneumoniae infection in pediatric patients. *Antimicrob Agents Chemother*. Aug 2013;57(8):4046-9. doi:10.1128/aac.00663-13

51. Kawakami N, Namkoong H, Saito F, Ishizaki M, Yamazaki M, Mitamura K. Epidemiology of macrolide-resistant Mycoplasma pneumoniae by age distribution in Japan. *J Infect Chemother*. Jan 2021;27(1):45-48. doi:10.1016/j.jiac.2020.08.006

52. Kenri T, Suzuki M, Sekizuka T, et al. Periodic Genotype Shifts in Clinically Prevalent Mycoplasma pneumoniae Strains in Japan. *Front Cell Infect Microbiol*. 2020;10:385. doi:10.3389/fcimb.2020.00385

53. Kim JH, Kim JY, Yoo CH, et al. Macrolide Resistance and Its Impacts on M. Pneumoniae Pneumonia in Children: Comparison of Two Recent Epidemics in Korea. *Allergy Asthma Immunol Res*. Jul 2017;9(4):340-346. doi:10.4168/aair.2017.9.4.340

54. Kim M, Choi JH, Kim YR, Kim C. Incidence and clinical features of communityacquired pneumonia

caused by macrolideresistant mycoplasma pneumoniae from adults in Jeju. Conference Abstract. *Respirology*. 2018;23:308. doi:10.1111/resp.13420_606

55. Kim YJ, Shin KS, Lee KH, Kim YR, Choi JH. Clinical Characteristics of Macrolide-Resistant Mycoplasma pneumoniae from Children in Jeju. *J Korean Med Sci*. Oct 2017;32(10):1642-1646. doi:10.3346/jkms.2017.32.10.1642

56. Kogoj R, Praprotnik M, Mrvič T, Korva M, Keše D. Genetic diversity and macrolide resistance of Mycoplasma pneumoniae isolates from two consecutive epidemics in Slovenia. *Eur J Clin Microbiol Infect Dis*. Jan 2018;37(1):99-107. doi:10.1007/s10096-017-3106-5

57. Koike C, Nakamura T, Inui S, et al. [Macrolide resistance and detection in Mycoplasma pneumoniae at Kansai Medical University Hirakata Hospital]. *Kansenshogaku Zasshi*. Nov 2011;85(6):652-7. doi:10.11150/kansenshogakuzasshi.85.652

58. Komatsu H, Tsunoda T, Inui A, Sogo T, Fujisawa T. Characteristics of hospitalized children infected with macrolide-resistant Mycoplasma pneumoniae. *Braz J Infect Dis*. May-Jun 2014;18(3):294-9. doi:10.1016/j.bjid.2013.09.004

59. Kurkela S, Puolakkainen M, Hokynar K, et al. Mycoplasma pneumoniae outbreak, Southeastern Finland, 2017-2018: molecular epidemiology and laboratory diagnostic lessons. *Eur J Clin Microbiol Infect Dis*. Oct 2019;38(10):1867-1871. doi:10.1007/s10096-019-03619-7

60. Lanata MM, Wang H, Everhart K, Moore-Clingenpeel M, Ramilo O, Leber A. Macrolide-Resistant Mycoplasma pneumoniae Infections in Children, Ohio, USA. *Emerg Infect Dis*. Jun 2021;27(6):1588-1597. doi:10.3201/eid2706.203206

61. Leal SM, Jr., Totten AH, Xiao L, et al. Evaluation of Commercial Molecular Diagnostic Methods for Detection and Determination of Macrolide Resistance in Mycoplasma pneumoniae. *J Clin Microbiol*. May 26 2020;58(6)doi:10.1128/jcm.00242-20

62. Lee E, Cho HJ, Hong SJ, Lee J, Sung H, Yu J. Prevalence and clinical manifestations of macrolide resistant Mycoplasma pneumoniae pneumonia in Korean children. *Korean J Pediatr*. May 2017;60(5):151-157. doi:10.3345/kjp.2017.60.5.151

63. Lee H, Choi YY, Sohn YJ, et al. Clinical Efficacy of Doxycycline for Treatment of Macrolide-Resistant Mycoplasma pneumoniae Pneumonia in Children. *Antibiotics (Basel)*. Feb 17 2021;10(2)doi:10.3390/antibiotics10020192

64. Lee JK, Choi YY, Sohn YJ, et al. Persistent high macrolide resistance rate and increase of macrolide-

© 2022 Kim K et al. *JAMA Network Open.*

resistant ST14 strains among Mycoplasma pneumoniae in South Korea, 2019-2020. *J Microbiol Immunol Infect*. Aug 26 2021;doi:10.1016/j.jmii.2021.07.011

65. Lee JK, Lee JH, Lee H, et al. Clonal Expansion of Macrolide-Resistant Sequence Type 3 Mycoplasma pneumoniae, South Korea. *Emerg Infect Dis*. Aug 2018;24(8):1465-1471. doi:10.3201/eid2408.180081

66. Li SL, Sun HM, Zhao HQ, et al. A single tube modified allele-specific-PCR for rapid detection of erythromycin-resistant Mycoplasma pneumoniae in Beijing. *Chin Med J (Engl)*. Aug 2012;125(15):2671-6.

67. Lin C, Li S, Sun H, et al. Nested PCR-linked capillary electrophoresis and single-strand conformation polymorphisms for detection of macrolide-resistant Mycoplasma pneumoniae in Beijing, China. *J Clin Microbiol*. Dec 2010;48(12):4567-72. doi:10.1128/jcm.00400-10

68. Liu X, Jiang Y, Chen X, Li J, Shi D, Xin D. Drug resistance mechanisms of Mycoplasma pneumoniae to macrolide antibiotics. *Biomed Res Int*. 2014;2014:320801. doi:10.1155/2014/320801

69. Liu Y, Ye X, Zhang H, et al. Antimicrobial susceptibility of Mycoplasma pneumoniae isolates and molecular analysis of macrolide-resistant strains from Shanghai, China. *Antimicrob Agents Chemother*. May 2009;53(5):2160-2. doi:10.1128/aac.01684-08

70. Liu Y, Ye X, Zhang H, et al. Characterization of macrolide resistance in Mycoplasma pneumoniae isolated from children in Shanghai, China. *Diagn Microbiol Infect Dis*. Aug 2010;67(4):355-8. doi:10.1016/j.diagmicrobio.2010.03.004

71. Liu Y, Ye X, Zhang H, Wu Z, Xu X. Rapid detection of Mycoplasma pneumoniae and its macrolide-resistance mutation by Cycleave PCR. *Diagn Microbiol Infect Dis*. Apr 2014;78(4):333-7. doi:10.1016/j.diagmicrobio.2013.12.002

72. Loconsole D, De Robertis AL, Mallamaci R, et al. First Description of Macrolide-Resistant Mycoplasma pneumoniae in Adults with Community-Acquired Pneumonia in Italy. *Biomed Res Int*. 2019;2019:7168949. doi:10.1155/2019/7168949

73. Loo LH, Soong HY, Maiwald M, Tee NW. Assessment of Genotypic Macrolide Resistance among Mycoplasma pneumoniae Infections in Children in Singapore. *Ann Acad Med Singap*. Jul 2017;46(7):290-292.

74. Lu CY, Yen TY, Chang LY, Liau YJ, Liu HH, Huang LM. Multiple-locus variable-number tandem-repeat analysis (MLVA) of macrolide-susceptible and -resistant Mycoplasma pneumoniae in children in Taiwan. *J Formos Med Assoc*. Oct 2020;119(10):1539-1545. doi:10.1016/j.jfma.2019.12.008

75. Lung DC, Yip EK, Lam DS, Que TL. Rapid defervescence after doxycycline treatment of macrolide-resistant Mycoplasma pneumoniae-associated community-acquired pneumonia in children. *Pediatr Infect Dis J*.

© 2022 Kim K et al. *JAMA Network Open.*

Dec 2013;32(12):1396-9. doi:10.1097/INF.0b013e3182a25c71

76. Ma Z, Zheng Y, Deng J, Ma X, Liu H. Characterization of macrolide resistance of Mycoplasma pneumoniae in children in Shenzhen, China. *Pediatr Pulmonol*. Jul 2014;49(7):695-700. doi:10.1002/ppul.22851

77. Matsubara K, Morozumi M, Okada T, et al. A comparative clinical study of macrolide-sensitive and macrolide-resistant Mycoplasma pneumoniae infections in pediatric patients. *J Infect Chemother*. Dec 2009;15(6):380-3. doi:10.1007/s10156-009-0715-7

78. Matsuda K, Narita M, Sera N, et al. Gene and cytokine profile analysis of macrolide-resistant Mycoplasma pneumoniae infection in Fukuoka, Japan. *BMC Infect Dis*. Dec 16 2013;13:591. doi:10.1186/1471-2334-13-591

79. Matsuoka M, Narita M, Okazaki N, et al. Characterization and molecular analysis of macrolide-resistant Mycoplasma pneumoniae clinical isolates obtained in Japan. *Antimicrob Agents Chemother*. Dec 2004;48(12):4624-30. doi:10.1128/aac.48.12.4624-4630.2004

80. Meyer Sauteur PM, Bleisch B, Voit A, et al. Survey of macrolide-resistant Mycoplasma pneumoniae in children with community-acquired pneumonia in Switzerland. *Swiss Med Wkly*. 2014;144:w14041. doi:10.4414/smw.2014.14041

81. Meyer Sauteur PM, Pánisová E, Seiler M, Theiler M, Berger C, Dumke R. Mycoplasma pneumoniae Genotypes and Clinical Outcome in Children. *J Clin Microbiol*. Jun 18 2021;59(7):e0074821. doi:10.1128/jcm.00748-21

82. Miyashita N, Akaike H, Teranishi H, Ouchi K, Okimoto N. Macrolide-resistant Mycoplasma pneumoniae pneumonia in adolescents and adults: clinical findings, drug susceptibility, and therapeutic efficacy. *Antimicrob Agents Chemother*. Oct 2013;57(10):5181-5. doi:10.1128/aac.00737-13

83. Miyashita N, Kawai Y, Ouchi K. Macrolide-resistant Mycoplasma pneumoniae in Japan. Conference Abstract. *Clinical Microbiology and Infection*. 2011;17:S252. doi:10.1111/j.1469-0691.2011.03558.x

84. Miyashita N KY, Akaike H, Ouchi K, Hayashi T, Kurihara T, Okimoto N; Atypical Pathogen Study Group. Macrolideresistant Mycoplasma pneumoniae in adolescents with community-acquired pneumonia. Conference Abstract. *BMC Infect Dis*. 2012;12:126.

85. Miyashita N, Oka M, Kawai Y, Yamaguchi T, Ouchi K. Macrolide-resistant Mycoplasma pneumoniae in adults with community-acquired pneumonia. *Int J Antimicrob Agents*. Oct 2010;36(4):384-5. doi:10.1016/j.ijantimicag.2010.06.009

© 2022 Kim K et al. *JAMA Network Open.*

86. Miyashita N, Sugiu T, Kawai Y, Yamaguchi T, Ouchi K. Macrolide-resistant mycoplasma pneumoniae in patients with community-acquired pneumonia in Japan. Conference Abstract. *Chest*. 2009;136(4)

87. Miyata I, McCormick DW, DeVincenzo JP, Miyairi I. Utility of a novel dichromatic real-time PCR detectionmethod of macrolide resistantmutations of Mycoplasma pneumoniae. Conference Abstract. *International Journal of Antimicrobial Agents*. 2013;42:S93. doi:10.1016/S0924-8579(13)70405-9

88. Morimoto K, Suzuki M, Yoshida LM, Minh LN, Ariyoshi K. High prevalence of macrolide resistant mycoplasma pneumoniae among middle age pneumonia in Japan. Conference Abstract. *Open Forum Infectious Diseases*. 2017;4:S585. doi:10.1093/ofid/ofx163.1532

89. Morinaga Y, Suzuki H, Notake S, et al. Evaluation of GENECUBE Mycoplasma for the detection of macrolide-resistant Mycoplasma pneumoniae. *J Med Microbiol*. Dec 2020;69(12):1346-1350. doi:10.1099/jmm.0.001264

90. Morozumi M, Chiba N, Okada T, et al. Antibiotic susceptibility in relation to genotype of Streptococcus pneumoniae, Haemophilus influenzae, and Mycoplasma pneumoniae responsible for community-acquired pneumonia in children. *J Infect Chemother*. Jun 2013;19(3):432-40. doi:10.1007/s10156-012-0500-x

91. Morozumi M, Hasegawa K, Kobayashi R, et al. Emergence of macrolide-resistant Mycoplasma pneumoniae with a 23S rRNA gene mutation. *Antimicrob Agents Chemother*. Jun 2005;49(6):2302-6. doi:10.1128/aac.49.6.2302-2306.2005

92. Morozumi M, Iwata S, Hasegawa K, et al. Increased macrolide resistance of Mycoplasma pneumoniae in pediatric patients with community-acquired pneumonia. *Antimicrob Agents Chemother*. Jan 2008;52(1):348-50. doi:10.1128/aac.00779-07

93. Morozumi M, Tajima T, Sakuma M, et al. Sequence Type Changes Associated with Decreasing Macrolide-Resistant Mycoplasma pneumoniae, Japan. *Emerg Infect Dis*. Sep 2020;26(9):2210-2213. doi:10.3201/eid2609.191575

94. Muto T, Nakamura N, Masuda Y, et al. Usefulness of Q-probe PCR in Children with Mycoplasma pneumniae infection. *Jpn J Infect Dis*. Jun 30 2021;doi:10.7883/yoken.JJID.2021.003

95. Nagita A, Muramatsu H, Hokama M, et al. Efficiency of the novel quenching-probe PCR method to detect 23S rRNA mutations in children with Mycoplasma pneumoniae infection. *J Microbiol Methods*. Feb 2021;181:106135. doi:10.1016/j.mimet.2021.106135

96. Nakamura Y, Oishi T, Kaneko K, et al. Recent acute reduction in macrolide-resistant Mycoplasma pneumoniae infections among Japanese children. *J Infect Chemother*. Feb 2021;27(2):271-276.

© 2022 Kim K et al. *JAMA Network Open.*

doi:10.1016/j.jiac.2020.10.007

97. Nummi M, Mannonen L, Puolakkainen M. Development of a multiplex real-time PCR assay for detection of Mycoplasma pneumoniae, Chlamydia pneumoniae and mutations associated with macrolide resistance in Mycoplasma pneumoniae from respiratory clinical specimens. *Springerplus*. 2015;4:684. doi:10.1186/s40064-015-1457-x

98. Okada T, Morozumi M, Tajima T, et al. Rapid effectiveness of minocycline or doxycycline against macrolide-resistant Mycoplasma pneumoniae infection in a 2011 outbreak among Japanese children. *Clin Infect Dis*. Dec 2012;55(12):1642-9. doi:10.1093/cid/cis784

99. Ouchi K. The nationwide survey of mycoplasma pneumoniae infection in children throughout Japan in recent 10 years. Conference Abstract. *Pediatric Pulmonology*. 2019;54:S106. doi:10.1002/ppul.24373

100. Pereyre S, Charron A, Hidalgo-Grass C, et al. The spread of Mycoplasma pneumoniae is polyclonal in both an endemic setting in France and in an epidemic setting in Israel. *PLoS One*. 2012;7(6):e38585. doi:10.1371/journal.pone.0038585

101. Pereyre S, Renaudin H, Charron A, Bébéar C. Clonal spread of Mycoplasma pneumoniae in primary school, Bordeaux, France. Letter. *Emerging Infectious Diseases*. 2012;18(2):343-345. doi:10.3201/eid1802.111379

102. Pereyre S, Touati A, Petitjean-Lecherbonnier J, Charron A, Vabret A, Bébéar C. The increased incidence of Mycoplasma pneumoniae in France in 2011 was polyclonal, mainly involving M. pneumoniae type 1 strains. *Clin Microbiol Infect*. Apr 2013;19(4):E212-7. doi:10.1111/1469-0691.12107

103. Peuchant O, Ménard A, Renaudin H, et al. Increased macrolide resistance of Mycoplasma pneumoniae in France directly detected in clinical specimens by real-time PCR and melting curve analysis. *J Antimicrob Chemother*. Jul 2009;64(1):52-8. doi:10.1093/jac/dkp160

104. Pouladi I, Mirnejad R, Rostampur S, Viesy S, Niakan M. Molecular Detection and Evaluation of ML-Resistance M. Pneumoniae Associated with Mutation in 23S RNA Gene among Iranian Patients with Respiratory Infections. *Rep Biochem Mol Biol*. Jul 2020;9(2):223-229. doi:10.29252/rbmb.9.2.223

105. Qu J, Chen S, Bao F, Gu L, Cao B. Molecular characterization and analysis of Mycoplasma pneumoniae among patients of all ages with community-acquired pneumonia during an epidemic in China. *Int J Infect Dis*. Jun 2019;83:26-31. doi:10.1016/j.ijid.2019.03.028

106. Qu J, Yu X, Liu Y, et al. Specific multilocus variable-number tandem-repeat analysis genotypes of Mycoplasma pneumoniae are associated with diseases severity and macrolide susceptibility. *PLoS One*.

© 2022 Kim K et al. *JAMA Network Open.*

2013;8(12):e82174. doi:10.1371/journal.pone.0082174

107. Rivaya B, Jordana-Lluch E, Fernández-Rivas G, et al. Macrolide resistance and molecular typing of Mycoplasma pneumoniae infections during a 4 year period in Spain. *J Antimicrob Chemother*. Oct 1 2020;75(10):2752-2759. doi:10.1093/jac/dkaa256

108. Rodriguez N, Mondeja B, Sardiñas R, Vega D, Dumke R. First detection and characterization of macrolide-resistant Mycoplasma pneumoniae strains in Cuba. *Int J Infect Dis*. Mar 2019;80:115-117. doi:10.1016/j.ijid.2018.12.018

109. Smith S, Adamson PJ, Sadlon TA, Gordon DL. Prevalence of macrolide-resistant Mycoplasma pneumoniae in South Australia. *Pathology*. Oct 2016;48(6):639-42. doi:10.1016/j.pathol.2016.06.004

110. Spuesens EB, Meijer A, Bierschenk D, et al. Macrolide resistance determination and molecular typing of Mycoplasma pneumoniae in respiratory specimens collected between 1997 and 2008 in The Netherlands. *J Clin Microbiol*. Jun 2012;50(6):1999-2004. doi:10.1128/jcm.00400-12

111. Dumke R, Von Baum H, Lück C, Jacobs E. Occurrence of macrolide-resistant Mycoplasma pneumoniae strains in Germany. Conference Abstract. *International Journal of Medical Microbiology*. 2009;299:80.

112. Sun H, Xue G, Yan C, et al. Changes in Molecular Characteristics of Mycoplasma pneumoniae in Clinical Specimens from Children in Beijing between 2003 and 2015. *PLoS One*. 2017;12(1):e0170253. doi:10.1371/journal.pone.0170253

113. Suzuki S, Konno T, Shibata C, Saito H. Low Incidence of Macrolide-Resistant Mycoplasma pneumoniae between April 2016 and March 2017 in Akita Prefecture, Japan. *Jpn J Infect Dis*. Nov 22 2018;71(6):477-478. doi:10.7883/yoken.JJID.2018.170

114. Suzuki Y, Itagaki T, Seto J, et al. Community outbreak of macrolide-resistant Mycoplasma pneumoniae in Yamagata, Japan in 2009. *Pediatr Infect Dis J*. Mar 2013;32(3):237-40. doi:10.1097/INF.0b013e31827aa7bd

115. Suzuki Y, Seto J, Shimotai Y, et al. Multiple-Locus Variable-Number Tandem-Repeat Analysis of Mycoplasma pneumoniae Isolates between 2004 and 2014 in Yamagata, Japan: Change in Molecular Characteristics during an 11-year Period. *Jpn J Infect Dis*. Nov 22 2017;70(6):642-646. doi:10.7883/yoken.JJID.2017.276

116. Tanaka T, Oishi T, Miyata I, et al. Macrolide-Resistant Mycoplasma pneumoniae Infection, Japan, 2008-2015. *Emerg Infect Dis*. Oct 2017;23(10):1703-1706. doi:10.3201/eid2310.170106

© 2022 Kim K et al. *JAMA Network Open.*

117. Uh Y, Hong JH, Oh KJ, et al. Macrolide resistance of Mycoplasma pneumoniae and its detection rate by real-time PCR in primary and tertiary care hospitals. *Ann Lab Med*. Nov 2013;33(6):410-4. doi:10.3343/alm.2013.33.6.410

118. Uldum SA, Bangsborg JM, Gahrn-Hansen B, et al. Epidemic of Mycoplasma pneumoniae infection in Denmark, 2010 and 2011. *Euro Surveill*. Feb 2 2012;17(5)doi:10.2807/ese.17.05.20073-en

119. Voronina EN, Gordukova MA, Turina IE, et al. Molecular characterization of Mycoplasma pneumoniae infections in Moscow from 2015 to 2018. *Eur J Clin Microbiol Infect Dis*. Feb 2020;39(2):257-263. doi:10.1007/s10096-019-03717-6

120. Wagner K, Imkamp F, Pires VP, Keller PM. Evaluation of Lightmix Mycoplasma macrolide assay for detection of macrolide-resistant Mycoplasma pneumoniae in pneumonia patients. *Clin Microbiol Infect*. Mar 2019;25(3):383.e5-383.e7. doi:10.1016/j.cmi.2018.10.006

121. Waites KB, Ratliff A, Crabb DM, et al. Macrolide-Resistant Mycoplasma pneumoniae in the United States as Determined from a National Surveillance Program. *J Clin Microbiol*. Nov 2019;57(11)doi:10.1128/jcm.00968-19

122. Waller JL, Diaz MH, Petrone BL, et al. Detection and characterization of Mycoplasma pneumoniae during an outbreak of respiratory illness at a university. *J Clin Microbiol*. Mar 2014;52(3):849-53. doi:10.1128/jcm.02810-13

123. Wang Y, Qiu S, Yang G, et al. An outbreak of Mycoplasma pneumoniae caused by a macrolide-resistant isolate in a nursery school in China. *Antimicrob Agents Chemother*. Jul 2012;56(7):3748-52. doi:10.1128/aac.00142-12

124. Wang Y, Xu B, Wu X, et al. Increased Macrolide Resistance Rate of M3562 Mycoplasma pneumoniae Correlated With Macrolide Usage and Genotype Shifting. *Front Cell Infect Microbiol*. 2021;11:675466. doi:10.3389/fcimb.2021.675466

125. Wang Y, Ye Q, Yang D, Ni Z, Chen Z. Study of Two Separate Types of Macrolide-Resistant Mycoplasma pneumoniae Outbreaks. *Antimicrob Agents Chemother*. Jul 2016;60(7):4310-4. doi:10.1128/aac.00198-16

126. Yin Y, Wang R, Zhuo C, et al. Epidemiological monitoring and antibiotic therapies of macrolide-resistant mycoplasma pneumoniae in Chinese patients with community-acquired pneumonia: A prospective multicenter surveillance study. Conference Abstract. *Open Forum Infectious Diseases*. 2017;4:S571. doi:10.1093/ofid/ofx163.1491

© 2022 Kim K et al. *JAMA Network Open.*

127. Whistler T, Sawatwong P, Diaz MH, et al. Molecular Characterization of Mycoplasma pneumoniae Infections in Two Rural Populations of Thailand from 2009 to 2012. *J Clin Microbiol*. Jul 2017;55(7):2222-2233. doi:10.1128/jcm.00350-17

128. Wu HM, Wong KS, Huang YC, et al. Macrolide-resistant Mycoplasma pneumoniae in children in Taiwan. *J Infect Chemother*. Aug 2013;19(4):782-6. doi:10.1007/s10156-012-0523-3

129. Wu PS, Chang LY, Lin HC, et al. Epidemiology and clinical manifestations of children with macrolide-resistant Mycoplasma pneumoniae pneumonia in Taiwan. *Pediatr Pulmonol*. Sep 2013;48(9):904-11. doi:10.1002/ppul.22706

130. Xiao L, Ratliff AE, Crabb DM, et al. Molecular Characterization of Mycoplasma pneumoniae Isolates in the United States from 2012 to 2018. *J Clin Microbiol*. Sep 22 2020;58(10)doi:10.1128/jcm.00710-20

131. Xin DL, Mi ZH, Han X, et al. [Application of nested PCR and sequencing technique to detect point mutations of the 23S rRNA gene of Mycoplasma pneumoniae]. *Zhonghua Er Ke Za Zhi*. Jul 2008;46(7):522-5.

132. Xin D, Mi Z, Han X, et al. Molecular mechanisms of macrolide resistance in clinical isolates of Mycoplasma pneumoniae from China. *Antimicrob Agents Chemother*. May 2009;53(5):2158-9. doi:10.1128/aac.01563-08

133. Xu C, Deng H, Zhang J, et al. Mutations in domain V of Mycoplasma pneumoniae 23S rRNA and clinical characteristics of pediatric M. pneumoniae pneumonia in Nanjing, China. *J Int Med Res*. Jun 2021;49(6):3000605211016376. doi:10.1177/03000605211016376

134. Xue G, Li M, Wang N, et al. Comparison of the molecular characteristics of Mycoplasma pneumoniae from children across different regions of China. *PLoS One*. 2018;13(8):e0198557. doi:10.1371/journal.pone.0198557

135. Xue G, Wang Q, Yan C, et al. Molecular characterizations of PCR-positive Mycoplasma pneumoniae specimens collected from Australia and China. *J Clin Microbiol*. May 2014;52(5):1478-82. doi:10.1128/jcm.03366-13

136. Yamada M, Buller R, Bledsoe S, Storch GA. Rising rates of macrolide-resistant Mycoplasma pneumoniae in the central United States. *Pediatr Infect Dis J*. Apr 2012;31(4):409-0. doi:10.1097/INF.0b013e318247f3e0

137. Yan C, Sun H, Lee S, et al. Comparison of Molecular Characteristics of Mycoplasma pneumoniae Specimens Collected from the United States and China. *J Clin Microbiol*. Dec 2015;53(12):3891-3. doi:10.1128/jcm.02468-15

© 2022 Kim K et al. *JAMA Network Open.*

138. Yan C, Yang H, Sun H, et al. Diversity in Genotype Distribution of Mycoplasma pneumoniae Obtained from Children and Adults. *Jpn J Infect Dis*. Jan 23 2020;73(1):14-18. doi:10.7883/yoken.JJID.2019.037

139. Yin YD, Wang R, Zhuo C, et al. Macrolide-resistant Mycoplasma pneumoniae prevalence and clinical aspects in adult patients with community-acquired pneumonia in China: a prospective multicenter surveillance study. *J Thorac Dis*. Oct 2017;9(10):3774-3781. doi:10.21037/jtd.2017.09.75

140. Yoo SJ, Kim HB, Choi SH, et al. Differences in the frequency of 23S rRNA gene mutations in Mycoplasma pneumoniae between children and adults with community-acquired pneumonia: clinical impact of mutations conferring macrolide resistance. *Antimicrob Agents Chemother*. Dec 2012;56(12):6393-6. doi:10.1128/aac.01421-12

141. Yu HX, Zhao MM, Pu ZH, Ju YR, Liu Y. A study of community-acquired Mycoplasma pneumoniae in Yantai, China. *Colomb Med (Cali)*. Jun 30 2018;49(2):160-163. doi:10.25100/cm.v49i2.3813

142. Zhang WZ, Zhang SJ, Wang QY, et al. Outbreak of macrolide-resistant mycoplasma pneumoniae in a primary school in Beijing, China in 2018. *BMC Infect Dis*. Oct 22 2019;19(1):871. doi:10.1186/s12879-019-4473-6

143. Zhao F, Liu G, Wu J, et al. Surveillance of macrolide-resistant Mycoplasma pneumoniae in Beijing, China, from 2008 to 2012. *Antimicrob Agents Chemother*. Mar 2013;57(3):1521-3. doi:10.1128/aac.02060-12

144. Zhao F, Li J, Liu J, et al. Antimicrobial susceptibility and molecular characteristics of Mycoplasma pneumoniae isolates across different regions of China. *Antimicrob Resist Infect Control*. 2019;8:143. doi:10.1186/s13756-019-0576-5

145. Zhao F, Liu J, Shi W, et al. Antimicrobial susceptibility and genotyping of Mycoplasma pneumoniae isolates in Beijing, China, from 2014 to 2016. *Antimicrob Resist Infect Control*. 2019;8:18. doi:10.1186/s13756-019-0469-7

146. Zhao H, Li S, Cao L, et al. Surveillance of Mycoplasma pneumoniae infection among children in Beijing from 2007 to 2012. *Chin Med J (Engl)*. 2014;127(7):1244-8.

147. Zheng X, Lee S, Selvarangan R, et al. Macrolide-Resistant Mycoplasma pneumoniae, United States. *Emerg Infect Dis*. Aug 2015;21(8):1470-2. doi:10.3201/eid2108.150273

148. Zhou Y, Wang J, Chen W, et al. Impact of viral coinfection and macrolide-resistant mycoplasma infection in children with refractory Mycoplasma pneumoniae pneumonia. *BMC Infect Dis*. Aug 26 2020;20(1):633. doi:10.1186/s12879-020-05356-1

© 2022 Kim K et al. *JAMA Network Open.*

149. Zhou Y, Zhang Y, Sheng Y, Zhang L, Shen Z, Chen Z. More complications occur in macrolide-resistant than in macrolide-sensitive Mycoplasma pneumoniae pneumonia. *Antimicrob Agents Chemother*. 2014;58(2):1034-8. doi:10.1128/aac.01806-13

150. Zhu M, Zhao J, Song L, Xu M, Ji J. Effects of 2063 locus gene mutation of 23S rRNA V region in MP pneumonia patients on macrolide drug resistance and DNA load. Article. *Acta Medica Mediterranea*. 2020;36(3):1715-1719. doi:10.19193/0393-6384_2020_3_269

© 2022 Kim K et al. *JAMA Network Open.*
